# Phenomenological Modeling of Antibody Response from Vaccine Strain Composition

**DOI:** 10.3390/antib14010006

**Published:** 2025-01-16

**Authors:** Victor Ovchinnikov, Martin Karplus

**Affiliations:** 1 Department of Chemistry and Chemical Biology, Harvard University, Cambridge, MA 02138, USA; 2 Laboratoire de Chimie Biophysique, ISIS, Université de Strasbourg, 67000 Strasbourg, France

**Keywords:** IgG, vaccination, simulation, influenza, hemagglutinin, coronavirus

## Abstract

The elicitation of broadly neutralizing antibodies (bnAbs) is a major goal of vaccine design for highly mutable pathogens, such as influenza, HIV, and coronavirus. Although many rational vaccine design strategies for eliciting bnAbs have been devised, their efficacies need to be evaluated in preclinical animal models and in clinical trials. To improve outcomes for such vaccines, it would be useful to develop methods that can predict vaccine efficacies against arbitrary pathogen variants. As a step in this direction, here, we describe a simple biologically motivated model of antibody reactivity elicited by nanoparticle-based vaccines using only antigen amino acid sequences, parametrized with a small sample of experimental antibody binding data from influenza or SARS-CoV-2 nanoparticle vaccinations. **Results**: The model is able to recapitulate the experimental data to within experimental uncertainty, is relatively insensitive to the choice of the parametrization/training set, and provides qualitative predictions about the antigenic epitopes exploited by the vaccine, which are testable by experiment. For the mosaic nanoparticle vaccines considered here, model results suggest indirectly that the sera obtained from vaccinated mice contain bnAbs, rather than simply different strain-specific Abs. Although the present model was motivated by nanoparticle vaccines, we also apply it to a mutlivalent mRNA flu vaccination study, and demonstrate good recapitulation of experimental results. This suggests that the model formalism is, in principle, sufficiently flexible to accommodate different vaccination strategies. Finally, we show how the model could be used to rank the efficacies of vaccines with different antigen compositions. **Conclusions**: Overall, this study suggests that simple models of vaccine efficacy parametrized with modest amounts of experimental data could be used to compare the effectiveness of designed vaccines.

## 1. Introduction

Infections by highly mutable viruses cause high mortality and morbidity around the world. For example, according to the CDC, between the years 2010–2014, influenza-associated deaths in the United States alone ranged from 12,000 (winter of 2011–2012) to 56,000 (winter of 2012–2013). The currently available flu vaccines need to be reformulated yearly because the high mutation rate of the circulating flu strains continuously renders preexisting immunity obsolete. Despite the yearly adjustments, the long time lag in producing the vaccine relative to the high rate of antigenic drift can result in a low level of protective immunity (10–60%) [1]. For example, in 2022, the seasonal influenza vaccine efficacy was estimated at 16% [2], significantly below the approximate threshold needed for herd immunity of 50% (assuming a basic viral reproduction number $R_{0}$ [3] of 2).

The COVID-19 pandemic resulted in rapid development of highly effective vaccines [4], with effectiveness of ≥90% against the original (Wuhan) strain [5,6]. However, mutations in the coronavirus spike protein [7], compounded by high transmission rates [3] and selective evolutionary pressure, exerted by vaccine-induced antibodies, caused the emergence of viral “escape” variants, against which the antibodies induced by the standard prime-boost vaccination regimen had much reduced efficacy, e.g., 67–70% against, e.g., the Omicron variant [8]. Such levels of efficacy are lower than the 80% requirement for herd immunity based on a conservative $R_{0}$ estimate of 5 [9].

The situation is more dire with HIV, which causes about 680,000 yearly deaths worldwide [10], and no effective vaccine is available, despite ongoing efforts [11].

The above statistics demonstrate that universal, ideally permanent, vaccines for highly mutable viral pathogens are desirable. One strategy for developing such vaccines focuses on the elicitation of broadly neutralizing antibodies (bnAbs), i.e., the Abs that are able to neutralize a broad range of antigen variants [12,13], in contrast to strain-specific antibodies, which typically dominate the adaptive immune response [14].

Universal vaccine candidates designed to elicit broad protection typically aim to present multiple epitopes to the immune system in the form of chimeric antigens harboring a patchwork of different epitopes [15], in the form of optimized antigenic cocktails [16,17], or as co-display of different antigens on nanoparticles [18,19,20,21].

An important step toward robust rational design of such vaccines is being able to predict properties of the antibody response to vaccination. Such methods could be used in comparisons of the modeled outcomes to different vaccination cocktails or regimens, to select those that predict the highest antibody breadth or potency.

Many models exist that simulate affinity maturation (AM) inside germinal centers using concepts from biology and physics (reviewed in Ref. [22]). In these models, Darwinian evolution, driven by random mutations and the selection of B-cell receptors (BCRs) on the basis of their affinity for antigen, generates high-affinity antibodies. However, most of the AM models are too approximate or coarse-grained to be able to evaluate specific vaccines [23,24] and thus provide only general design guidelines (e.g., regarding efficacy of single-antigen vs. cocktail vaccination [25,26], or regarding the effects of binding valency or antigen concentration [24]). Although advances in computational methods and speed are enabling modeling of BCR/antigen interactions in all-atom detail [27], such models are still too expensive to be used for generating meaningful statistics.

Here, we propose a simple biologically motivated phenomenological model for reactivity to antigen variants of post-vaccination sera elicited by nanoparticle vaccines with prescribed antigenic sequences. The key assumptions in the models are that (i) antibody reactivity is higher when the test strain—that against which reactivity is measured—is similar to the strains in the vaccine, and (ii) different amino acid residues make different contributions to the overall binding titer; the contributions are represented in the form of residue weights, optimized by fitting to experimental vaccination data for influenza [20] or SARS-CoV-2 [21]. Residues with high weights can be interpreted as being involved in antibody binding, which can be tested by experimental mutagenesis to assess model accuracy. In addition to parametrization using experimental data, the model requires only the antigenic sequences, so that structural information, which could be difficult or time-consuming to obtain, is not needed. We conclude with an example of how the model could be used to compare the efficacies of different antigen vaccination cocktails.

## 2. Materials and Methods

### 2.1. Model Overview

Our modeling approach is motivated by the recent promise of nanoparticle vaccines in eliciting cross-reactive immunity [18,19,21,28]. We developed the model to be especially predictive if the elicited immunity is composed of cross-reactive antibodies, rather than only of multiple strain-specific antibodies. More specifically, if a particular B-cell receptor (BCR) has significant affinities for multiple strains in the cocktail via the same general epitope or region on the antigen, we assume that the overall selection pressure acting on the corresponding B-cell during affinity maturation (AM) will be the average of the individual (strain-specific) selection pressures. As the main result of AM is to increase the binding affinity of BCRs to their cognate antigen(s) by improving the structural complementarity of the BCR paratope to the epitope via interfacial residue composition matching [27], we assume that the effective evolutionary driving force is directed toward the epitope with the “average” composition. As the structures of the antigens are generally unknown, we model the effect of the average structure through the average sequence, denoted above by $\bar{V}$. If most of the Abs elicited by vaccination arise from such BCRs, then we would expect the model to recapitulate the experimental data [20,21]. We would also expect such Abs to be cross-reactive in the antigenic “subspace” spanned by the antigens that are present in the vaccine. We note that the AM process assumed above is similar to the idea of “frustration” [23,29], whereby different co-administered antigens exert conflicting selection pressures on BCR evolution, resulting in some averaged or compromise driving force. We caution that the above justification for the model is somewhat limited by our use of a single set of residue weights W. Although the model is generalizable to multiple weight vectors, which could be interpreted as, e.g., quantifying the contributions of distinct antigen epitopes, more complex models would require larger experimental datasets, and are left to future studies. Possible modifications to the modeling approach are discussed in the Supporting Material.

### 2.2. Sequence Coordinate Notation

Let $\hat{S}\in M_{N_{s}\times N_{r}}$ denote a multiple sequence alignment (MSA) matrix of $N_{s}$ antigen sequences, with each row $S_{i}$ of $\hat{S}$ corresponding to a unique sequence *i* of length $N_{r}$, possibly with gaps to facilitate the MSA. $\hat{S}$ is represented by the standard 20-amino-acid alphabet (*A*) and the gap symbol ($G\equiv^{\prime }{-}^{\prime }$).

**Figure 1:**
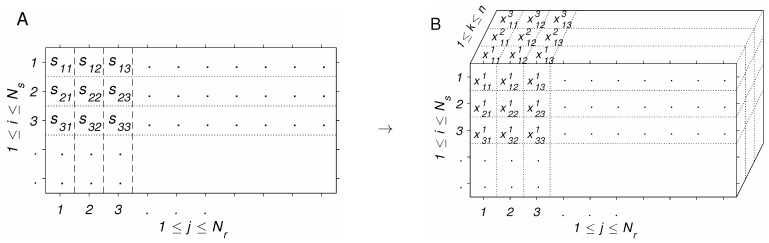
Illustration of the amino acid encoding procedure [31]. (**A**): Multiple alignment $\hat{S}$ of $N_{s}$ sequences, each having $N_{r}$ residues, including gaps, with $s_{ij}\in \{A,C,D,E,F,G,H,I,K,L,M,N,$$P,Q,R,S,T,V,W,Y{,}^{\prime }{-}^{\prime }\}$. (**B**): Embedded alignment $\hat{X}$; each residue type in $\hat{S}$ shown in A is associated with a 3-dimensional real-valued vector $x_{ij}$=$\left\{x_{ij}^{1},x_{ij}^{2},x_{ij}^{3}\right\}$, which is interpreted as Cartesian coordinates of the residue.

To be able to perform standard mathematical operations on $\hat{S}$, we embed $\hat{S}$ in an *n*-dimensional (nD) vector space, as follows. To each residue $s_{ij}$ at position *j* in $S_{i}$, we assign a vector $x_{ij}\in R_{n}\equiv x\left(s_{ij}\right)$ (see Figure 1). A number of such embeddings (also called encodings [30]) can be found in the literature [30,31,32,33], which have been used in machine learning models of sequence–activity relationships [33,34]. We note that the inverse map s(x) for arbitrary x is not needed in our study, because we do not create new sequences. However, such an inverse could be defined as a suitable projection onto the set of allowed residue coordinates, e.g.,

(1) $$s\left(x\right)=\arg\min_{s\in A\cup G}∥x-x\left(s\right)∥,$$

as conducted in Ref. [34].

Previous studies have found that the choice of embedding can have significant effects on model quality [30]. In the present calculations, we found that the simple 3D embedding model of Grantham [31], which is based on amino acid physico-chemical properties, produces model fits that approximate IgG data to within the experimental error, with high Pearson correlation coefficients (≃0.8–0.9) (see *Results*). The Grantham model uses three amino acid properties that show strong individual correlations with evolutionary residue exchangeability, (1) the atomic mass ratio of noncarbon to carbon atoms in the side chain, (2) residue polarity, and (3) molecular volume. Euclidean distances between amino acids computed by treating the normalized numerical values for each residue as orthogonal components show a Spearman rank (anti) correlation of −0.765 with residue substitution frequencies [31]. (The correlation is negative because residues that show greater exchangeability are modeled to have a smaller separation distance.) For completeness, we also tried a higher-dimensional (5D) embedding as per Atchley et al. [32]. These authors used a more sophisticated approach, first manually selecting 54 out of 494 amino acid attributes, and applying factor analysis to compute five factors as linear combinations of the original attributes. The factor scores are analogous to displacements along principal components in principal component analysis (PCA). In our calculations, the Atchley encoding gave slightly inferior agreement between modeled and experiment IgG (see *Results*). For simplicity, all gap positions in $\hat{S}$ are assigned the zero vector; i.e., we did not attempt to optimize the MSA representation of amino acid deletions. In the remainder of this section, we use the 3D embedded coordinates $x_{ij}^{k}$ with *k* = 1,2,3 to represent antigen residues in the MSA.

### 2.3. Distance to Average Strain IgG Model

Let $\hat{X}$ be the embedded sequence alignment as described above. We assume that a subset of *v* strains $V\equiv \{i_{1},i_{2},...i_{v}\}$ from $\hat{S}$ has been chosen as the vaccination cocktail $\hat{V}$, and let $\bar{V}$ be the average cocktail sequence. We use a simple average, but a weighted average could also be used, e.g., if the strains in the cocktail had different concentrations. We also note that additional (as yet unknown) strains can readily be added to the alignment *a posteriori*, i.e., after the model has been parametrized.

For any (test) sequence $T\in \hat{X}$, the IgG titer against T elicited by the cocktail $\hat{V}$ is modeled as

(2) $$IgG^{M}\left(\hat{V},T\right)\equiv f\left(d\left(\bar{V},T;W\right)\right),$$

where *d* is the weighted Euclidean distance between $\bar{V}$ and T with weights $W\in M_{1\times N_{r}}$ (specified below), i.e.,

(3) $$\begin{array}{cc} d(\bar{V},T;W)\; & ={\left\{\sum_{j=1}^{N_{r}}w_{j}^{2}{∥t_{j}-{\bar{v}}_{j}∥}^{2}\right\}}^{1/2}\\ \; & ={\left\{\sum_{j=1}^{N_{r}}w_{j}^{2}\sum_{k=1}^{n}{\left(t_{j}^{k}-{\bar{v}}_{j}^{k}\right)}^{2}\right\}}^{1/2} \end{array}$$

and

(4) $$f\left(d;\alpha ,\beta \right)=\frac{1}{\alpha +d^{\beta }}$$

is the sequence similarity function, with parameters α and β to be optimized below. Equation (2) assumes that IgG titers are determined by the similarity between the test strain and the *average* vaccination strain in the cocktail. The residue weights W, as well as α and β, are optimized by fitting to experimental IgG titers, as will be described at the end of this section. The reason for the rather ad hoc functional form of *f* is that it is one of the simplest ansätze that embodies an inverse relationship between distance and similarity, with sufficient flexibility for fitting through the coefficients α and β. While alternative functional forms are possible, e.g., f=αexp(−βd), and could be explored in the future, the fact that the majority of our modeled titers are within experimental uncertainty (see *Results*) suggests that the present choice is sufficient.

### 2.4. Model Parameter Optimization

**Figure 2:**
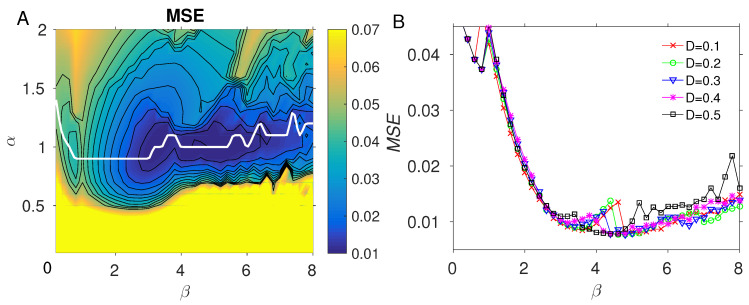
Parameter optimization (α,β) in the strain similarity function Equation (4) applied to influenza data [20] using a diffusion constant *D* = 0.4. (**A**): A 2D scan of the (α,β) landscape; the white line corresponds to a cubic spline curve through the minimum values of the mean squared error (MSE) over the range of α at each β; (**B**): MSE corresponding to the white line in A plotted in 1D for several values of the diffusion constant *D*.

Fitting the model to experimental data involves optimization of the parameters α, β and the residue weights W. Since the 2D parameter landscape of (α, β) was discovered to have multiple local minima, we performed a 2D scan of the parameter space to find optimal values (see Figure 2A). However, optimization of residue weights W in this fashion would be infeasible because of the large number of residues (∼550 for the influenza hemagglutinin MSA). Therefore, the weights were optimized using the steepest gradient descent, as follows.

Let $IgG^{E}\left(\hat{V},T\right)$ denote the experimental ELISA titer against test antigen T obtained using sera from animals vaccinated with cocktail $\hat{V}$ [20,21], and let $\delta IgG^{E}\left(\hat{V},T\right)$ denote the corresponding experimental uncertainty. The weighted squared error (WSE) of the antigen test set $\hat{T}$ is defined as

(5) $$\epsilon_{\hat{V},\hat{T}}^{2}=\sum_{T\in \hat{T}}{\left[\frac{IgG^{M}\left(\hat{V},T\right)-IgG^{E}\left(\hat{V},T\right)}{\delta IgG^{E}\left(\hat{V},T\right)}\right]}^{2},$$

where the dependence on W enters via Equation (2), and the subscripts on $\epsilon^{2}$ make explicit the dependence on $\hat{V}$ and $\hat{T}$, which will be omitted below for brevity. Starting from uniform initial weights W = 0.1, and values for (α,β) corresponding to the current position in the 2D scan (discussed further below), the weights are updated according to

(6) $$\begin{array}{cc} \; & W^{*}=W^{n}-\tau \nabla_{W}\epsilon^{2}+D\nabla^{2}W^{n},\\ \; & W^{n+1}=\max\left\{W^{*},0\right\}, \end{array}$$

where τ is the steepest descent step, $\nabla_{W}\left(\cdot \right)$ denotes gradients with respect to W, $\nabla^{2}W^{n}$ is the discrete analog of the 1D Laplacian of $W^{n}$ with respect to the residue position in the MSA, and *D* is an empirically selected diffusion constant. In the above, the diffusion term is added as a type of regularization, to enforce smoothness of W as a function of residue position. This approach reduces overfitting to experimental data, and also has a physical motivation: we can interpret the residues with high weights as those that contribute to antibody binding. The diffusion regularization term ensures that residues that are close to each other do not make vastly different contributions to binding. The sensitivity of the fits to the value of *D* is relatively minor, as will be shown below. The discrete Laplacian in our computer implementation of Equation (6) is

(7) $$\nabla^{2}w_{i}^{n}\equiv \left\{\begin{array}{cc} w_{i-1}^{n}-2w_{i}^{n}+w_{i+1}^{n}, & i=2,\cdots ,N_{r}-1\\ 0, & i=1,N_{r}, \end{array}\right.$$

which is equivalent to the finite difference of the second derivative computed to second order. The last line of Equation (6) ensures that W are non-negative at every step.

The expression for the gradient $\nabla_{W}\epsilon^{2}$ is obtained from Equations (2)–(4), i.e.,

(8) $$\begin{array}{cc} \frac{\partial \epsilon^{2}}{\partial w_{k}} & =\sum_{T\in \hat{T}}\left[\frac{IgG^{M}\left(\hat{V},T\right)-IgG^{E}\left(\hat{V},T\right)}{\delta IgG^{E}{\left(\hat{V},T\right)}^{2}}\right]\\ \; & \times 4f^{\prime }\left(d^{2}\right)w_{k}∥{\bar{v}}_{k}-t_{k}∥, \end{array}$$

where we wrote *f* as a function of $d^{2}$ rather than *d* to avoid the singularity of $\nabla_{W}d$ at *d* = 0.

The above error minimization equations are written under the assumption of a particular vaccination cocktail $\hat{V}$. However, we optimized a single set of weights for all of the vaccination cocktails in Ref. [21] or Ref. [20] simultaneously. In that case, the total squared error (and its gradients) are the sums of those corresponding to the individual vaccinations.

The results of optimizing the similarity function parameters (α,β) in Equation (4) are illustrated in Figure 2 for fitting to influenza data [20]. Figure 2A shows a 2D discrete scan of the parameter space (α,β)∈{0,0.1,0.2,⋯,2}×{0,0.2,0.4,⋯,8}. For each grid point, the weights W were reoptimized according to Equation (6) starting from $W^{0}$ = 0.1, and iterating for $n_{max}$ = 3000 steps with τ = 0.03, diffusion constant *D* = 0.4, and the mean squared error of the fit computed from Equation (5) and averaged. The scan shows a broad, irregularly shaped minimum for α≃ 1, β≳ 3); however, for β≳ 5, the optimization landscape shows oscillatory behavior, suggesting that the model becomes sensitive to parameters, or that the gradient descent step could be too large in this region. However, the 1D plot through the minimum at each β value (Figure 2B) shows that the global minimum lies within the region β∈(3,5), and suggests that further parameter space optimization or exploration are not necessary. We repeated the optimization for multiple values of *D* (Figure 2B) and observed that the error minimum and location are not very sensitive to the choice of *D*; however, for D≳0.5, we observed increased ruggedness, as can be seen for *D* = 0.5 (black line in Figure 2B). For the model calculations in *Results*, we used the value *D* = 0.49, which results in a relatively smooth distribution of the residue weights, as shown in the *Results* (e.g., Figure S4). We also noticed that the weights optimization could be performed with fewer iterations and at higher τ, with values up to 0.04, if the optimized weights were taken to be those that minimize the error over the *entire* optimization history (not just the final $W^{n}$ in Equation (6)), i.e.,

(9) $$W_{opt}=\arg\min_{0\leq n\leq n_{max}}\epsilon^{2}\left(W^{n}\right).$$

Parameter optimization for coronavirus data was very similar. The number of weights W to optimize was equal to the number of residue positions in the underlying MSA, which are 549 and 273 for influenza and coronavirus, respectively, subject to the constraints of non-negativity and diffusional regularization described above.

The parameters used to obtain the data in *Results* are given in Table 1. Calculations were performed on x86_64 AMD computers running Linux using Matlab [35] and GNU Octave [36], which is an open source program syntactically compatible with Matlab. A typical calculation to computed optimized weights and titer fits took about a minute on a single processor. Discrete scans of the (α,β) landscape required 800–1000 such calculations, taking about 14–16 h. Molecular structures for the visualization of weights were rendered using Visual Molecular Dynamics [37].

### 2.5. Principal Component Analysis

To visualize the landscape of influenza hemagglutinin (HA) sequences, and to aid in the selection of hypothetical vaccine strains, we used principal component analysis (PCA) in embedded coordinates $\hat{X}$ defined above. Briefly, sequences of avian, swine, and human influenza type A HA proteins spanning the years 1918–2019 and subtypes 1–18 were downloaded from the NIH influenza research database [38], clustered, and sampled so that no two retained sequences were more than 97% identical. The sequences were multiply aligned, and embedded in the 5D vector space of Atchley et al. [32], which was found to give a good visual separation of the HA subtypes in the 2D landscape (see *Results*).

The covariance matrix of sequence differences was computed as

(10) $$C_{jk}^{pq}=\langle \left(x_{ij}^{p}-{\langle x_{ij}^{p}\rangle }_{i}\right)\langle {(x_{ik}^{q}-{\langle x_{ik}^{q}\rangle }_{i}\rangle }_{i}$$

where the angle brackets represent averages over sequences (rows in the MSA), which are indexed by *i*; *j* and *k* denote residue positions, and *p* and *q* denote embedded coordinate components.

The covariance matrix was diagonalized to yield the diagonal eigenvalue (EV) matrix Λ and eigenvectors (principal components; PCs) *U*,

(11) $$C=U\Lambda U^{-1}.$$

The coordinate projection of any sequence *s* onto any PC vector, e.g., ${PC}_{l}^{q}$ is computed as

(12) $$PC_{l}^{q}=\sum_{j=1}^{N_{res}}\sum_{p=1}^{5}U_{lj}^{q}\left(x{\left(s\right)}_{j}^{p}-{\langle x_{ij}^{p}\rangle }_{i}\right),$$

where x(s) are the embedded coordinates of *s* after it has been added to the MSA. The double index on $PC_{l}^{q}$ is retained for notational convenience; in practice, we sort the PCs in the order of decreasing EVs and plot projections onto the two PCs corresponding to the highest EVs. The PCA was implemented in Matlab [35].

## 3. Results

First, we show that the model described in *Methods* reproduces measurements of post-vaccination mouse sera binding to panels of influenza [20] and SARS-Cov-2 [21] antigens to within experimental error. The virus strains and vaccine compositions are given in Table 2 and Table 3, for flu and coronavirus, respectively. Alignments of the antigen sequences and pairwise antigen similarity matrices are provided in supporting text, and in Figures S10 and S11.

**Figure 3:**
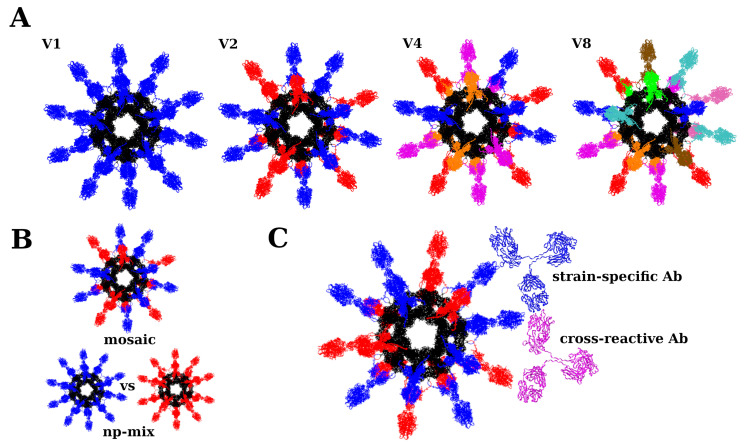
Illustration of the nanoparticles used in the vaccinations modeled here [20,21]. The nanoparticles are drawn in black, and the antigens are in color, with different colors indicating different strains. (**A**): Nanoparticles corresponding to the mosaic vaccines V1, V2, V4, and V8 in Table 2. (**B**): Mosaic vaccine (top) vs. nanoparticle mixture vaccine (bottom). (**C**): Hypothetical elicitation of strain-specific Abs (blue Ab binding to blue antigens) vs. cross-reactive bnAbs (purple Ab binding to blue and red antigens) by the mosaic vaccine via different Fabs.

Our focus is on mosaic nanoparticles (nps)—those that display the same protein antigen but from different strains—because such vaccines appear to elicit sera enriched in bnAbs [18,19,21,28] (see Figure 3 for an illustration of the nanoparticles used in the vaccinations). Fits to the titers for mixtures of homogeneous influenza nps are shown in the Supporting Material. Model performance is summarized in Table 4 and Table 5 for influenza and coronavirus, respectively.

### 3.1. Influenza Results

**Figure 4:**
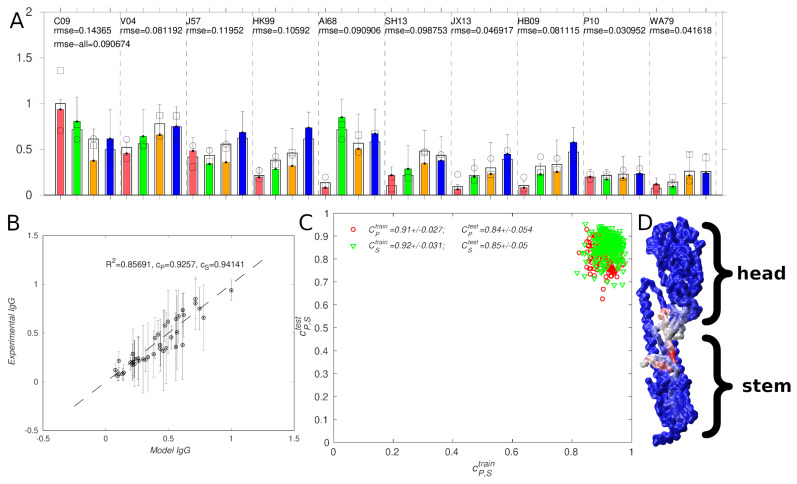
Comparison of model results with the experimental IgG titers for influenza [20]. (**A**,**B**): best fit to experiment; in (**A**), the colors correspond to experimental data for vaccines in Table 2 (red ■ V1, green ■ V2, orange ■ V4, blue ■ V8), and the black outer bars are model values. The black unfilled circles and squares correspond to model titers computed after setting to zero the residue weights in the HA stem and HA head, respectively (see text). (**C**): Comparison of 252 possible fits in which 5 strains were used for fitting (training) and 5 for testing (red ∘: $C_{P}$, green ▿: $C_{S}$). (**D**): Influenza hemagglutinin (PDB ID: 3LZG [39]) monomer colored by model weights using the color map blue-gray-red (corresponding to low-medium-high).

In Figure 4 we show the model results parametrized and tested using the the influenza vaccination data. Panels A and B show the optimized fits of the corresponding model to the IgG titers measured as area-under-curve (AUC) 21 days after vaccinating mice with mosaic nps [20].

Overall, the model shows a good fit to the average IgG titers (rmse = 0.09, $c_{P}$ = 0.93, $c_{S}$ = 0.94), where rmse is the root-mean-squared error, and $c_{P}$ and $c_{S}$ are the Pearson correlation and Spearman rank coefficients, respectively. Notably, the rmse of the corresponding experimental IgG error was 0.23, which was higher than that for the fit. The experimental error bars correspond to the standard deviations (σ) of the titer measurements; because there were four measurements of each titer, they also correspond to 2σ of the mean IgG, or the 95% confidence range. Panels A and B in Figure 4 show that all of the modeled titers for both models are within the corresponding error range.

To evaluate the predictive ability of the model, we performed the fitting procedure using three strategies. First, we carried out the fitting (or training) procedure with half of the strains (five), and used the remaining five strains to evaluate the fits. To eliminate the training/test set selection bias, we considered all 105 = 252 possible strain partitions. Panel C of Figure 4 shows scatter plots of the Pearson and Spearman correlations computed over the training vs. test sets, and the correlation statistics are summarized in Table 4 (section I). The correlation for the training set ($c_{P}∼$ 0.91) was higher than that for the test set ($c_{P}∼$ 0.84). This was expected, because the training (but not the test) set was used in the optimization/fitting. However, because the correlations were rather high even for the test set, it appears that overfitting to the training data was minor. Further, comparing the distributions of the correlation coefficients in terms of the σ, the differences were not significant even to within 1σ or 68% confidence.

Second, we performed the fitting procedure using data from two of the four vaccine titers, corresponding to 42 = 6 possible partitions. The results are summarized in Table 4 (section II). They are similar to those for the previous case, with the main difference being higher statistical errors in the correlation coefficients, as can also be seen from scatter plots of the Pearson and Spearman correlations in Figure S5.

In the third check, we removed the titers corresponding to vaccine V8 from the experimental dataset, and trained the models using 70% of the remaining data (i.e., 7 out of 10 strains). Taking 70%, rather than 50%, of the remaining data implies that the fraction of the training set relative to the size of the entire experimental sample is 0.7 × 0.75 = 0.525, which is roughly consistent with the 50% used in the first two cases above. The V8 vaccine titers were chosen as a "holdout" set because the V8 vaccine has some component strains that were not present in each of the remaining vaccines (Table 2), making it a nontrivial test case of a possible novel vaccine. Overall, the correlation coefficients were comparable to those from the previous two tests, the main difference being somewhat higher *p*-values, indicating lower statistical significance (see column 6 in Table 4). On average, the *p*-values correspond to greater than 95% significance, but the model parametrized using the Atchley encoding produced instances of particularly low statistical significance (the highest *p*-value of 0.77, which is why we favor using the Grantham encoding). The low-significance values were present despite the high Pearson correlation coefficient for the training set (≥0.9), suggesting that, in these cases, the models were overfit to the training data. To generate a single computational prediction for each of the measurements in the holdout set without model selection bias, the computed titers from all of the models corresponding to the different train/test partitions were averaged prior to computing correlations in Table 4 (holdout columns). These predictions generally produced somewhat lower correlation coefficients, in the range 0.63–0.89 with *p*-values ≤ 0.053 (see columns 7 and 8 in Table 4), still indicating satisfactory performance. Scatter plots corresponding to Table 4 section III are provided in Figure S8.

Overall, from Figure 4 panels A-C and Table 4, we can conclude that the model recapitulates the IgG titer data from the influenza nanoparticle vaccinations of Cohen et al. [20] with reasonable accuracy; the model gave better results for the mosaic vaccine dataset with the Grantham [31] encoding.

As described in *Methods*, a feature of the present model is that the residue weights W are interpretable as reflecting contributions to antibody binding. For example, we hypothesize that residues with high weights are involved in antibody/antigen binding. If true, the present models could aid in the identification of antigenic epitopes. To investigate this possibility, in panel D of Figure 4 we show the structure of an H1 hemagglutinin (HA) monomer [39] colored by residue weights; the weights are also plotted as a function of residue position in Figure S4. In the figures, the highest weights correspond to the upper portion of the HA stem, which is known to be targeted by several bnAbs [12]. In addition, the figure shows a residue patch along the top inner portion of the HA head, which is in the vicinity of the occluded epitope found by Watanabe et al. [40]. However, we note that the portion of the HA stem corresponding to the HA2 subunit does not contribute to binding in the above models, as the residue weights are zero in that region (see Figure S4A). Thus, the model may be too simple or approximate to identify all relevant binding epitopes. We note that, to actually map the epitopes to which the vaccination sera react would require antibody competition or epitope mutagenesis experiments. As the IgG model is nonlinear, we cannot directly separate the total IgG titer into relative contributions from the HA head and the HA stem. However, we can alternately set the residue weights in the head or the stem region to zero, renormalize the weights (to approximately preserve the total titer), and recompute the modeled titers. The results are shown in Figure 4A, with unfilled circles and squares corresponding to head-only and stem-only weights. The modeled values are generally in worse agreement with the experiment, demonstrating that residue weights in both the head and the stem region are important. The overall rmse between the modeled and experimental results in both cases increased by ∼50%, being somewhat larger in the head-only case.

### 3.2. Coronavirus Results

**Figure 5:**
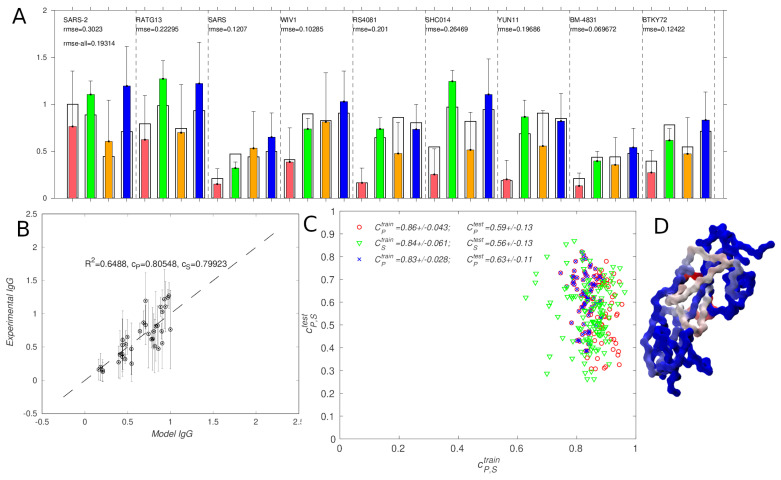
Comparison of Model 1 results with the experimental IgG titers for coronavirus [21]. (**A**,**B**): Best fit to experiment; in (**A**), the colors correspond to experimental data for vaccines in Table 3 (red ■ V1, green ■ V4A, orange ■ V4B, blue ■ V8), and the black outer bars are model values. (**C**): Comparison of 126 possible fits in which 5 strains were used for fitting (training) and 4 for testing (red ∘: $C_{P}$, green ▿: $C_{S}$, blue ×: $C_{P}$ for training sets that include SARS-2). (**D**): SARS-CoV-2 receptor binding domain (RBD), colored by model weights using the color map blue-gray-red (corresponding to low-medium-high); PDB ID: 6VXX [41].

Figure 5 shows the model results parametrized and tested using coronavirus vaccination IgG titers measured as AUC 14 days after vaccination with mosaic nps [21]. The main difference between these data and the influenza data is that model produces a somewhat worse fit (rmse = 0.19, $c_{P}$ = 0.81, $c_{S}$ = 0.80), which can be seen in panels A and B of Figure 5. Nonetheless, most of the modeled values in Figure 5A,B are within the experimental error bar. The experimental rmse in the titers, with ten replicates per value, was 0.36 [21]; the 95% confidence limit for the average value would correspond to a reduction in the error bar by a factor of only $2/\sqrt{10}≃0.63$, giving 0.23, which is comparable to the rmse for the model. Quantitatively, we evaluated the model performance using three strategies, as described for influenza. First, we compared the distributions of the correlations obtained when the titer data were split into a training set with five antigen strains, and the remaining four strains were used for testing. There were 95=126 such set pairs, corresponding to the scatter plot in Figure 5 panel C.

As before, the correlations for the training sets ($c_{P}∼$ 0.86 and $c_{S}∼$ 0.84) were higher than those for the test set ($c_{P}∼$ 0.59 and $c_{S}∼$ 0.56, respectively). These differences were somewhat higher that those for the influenza data, suggesting that there was more overfitting to the training set for the coronavirus data. Although, on the average, the *p*-value for the Pearson correlation coefficient was 0.039, the maximum value was 0.25, indicating that some correlation values were not very significant (see Table 5 section I). This was caused, in part, by the small size of the dataset, which had only 36 IgG values in total (Figure 5).

Next, we split the experimental data along vaccine groups, choosing two groups for training and two for testing, for a total of six possible splits, as was performed for the influenza data. The results were similar to the first test above ($c_{P}^{test}∼$ 0.65), and the maximum *p*-value was somewhat lower (0.038; Table 5 section II). The correlation scatter plots are shown in Figure S6.

Finally, we evaluated the model on three-way data splits, in which all IgG titers corresponding to vaccine V8 were set aside as a holdout set, and two-thirds of the remaining data (six out of nine antigen strains) were used for training. Using two-thirds corresponded to 50% of the total data, consistent with the above two checks and with the testing procedure for influenza. Applying the models to the holdout set and averaging the computed titers over the models, as performed above for influenza, resulted in the Pearson correlation of 0.8 (*p*-values ≤ 0.026; Table 5 section III). This result appears satisfactory, especially given the small amount of training data. The correlation scatter plots for this case are shown in Figure S9.

Although the somewhat greater degree of overfitting mentioned above suggests that the model does not perform as well as for the influenza np dataset (compare Figure 4 and Figure 5’s panels C), the $c^{train}$ vs. $c^{test}$ scatter plots show that there are several model instances in which the correlation on both the training and test sets was around ∼0.8. Moreover, the fact that the average model titers over all of the trained models applied to the holdout set correlate with the experiment to ∼0.8 indicate that, even with the small size of the dataset, it is possible to obtain a satisfactory model.

It is noteworthy that a large discrepancy between the model and experiment was found for the IgG titers obtained after vaccination with only the SARS-Cov-2 receptor-binding domain (RBD) antigen, measured against the same antigen (Figure 5 panel A, first bar from the left). As the test strain and the vaccine strain are identical, one would expect to see strong binding between the vaccination sera and the antigen. However, some serum samples showed no detectable binding in the experiment [21], which caused the high error bar and the lower average titer, compared with vaccines V4A (green bars) and V8 (blue bars) (see Table 3 and Figure 5).

This result seems at odds with the main assumption made in our model, i.e., that sera reactivity increases with the similarity of the test strain to the vaccination strain(s) (Equation (2)), and, in part, explains the worse fit and prediction quality. Other strains for which the discrepancies between the model and experimental IgG values are large are RS4081, SHC014, and YUN11, especially when measured with respect to the sera of vaccine V4B (orange bars in Figure 5). However, none of those strains are components of V4B (see Table 3). Therefore, the titer differences were, in principle, justifiable as modeling errors due to strain mismatch, rather than only due to non-binding experimental samples. In contrast, for the SARS-2 case, the match between the vaccine and test strains was 100%, which is why a low titer appeared unphysical. Although it might be tempting to consider the non-binding samples as erroneous or as outliers, we do not have sufficient evidence to do so; e.g., multiple different vaccination sera samples had very low titers, which cause high error bars [21]. Unfortunately, there are no experimental data that could provide a mechanistic explanation for the low binding titers to the vaccinating antigen in the SARS-2 group. It seems possible that different animals developed sufficiently different antibody profiles to the vaccinating antigen to explain the differences, but discriminating between a multi-epitope model and the present model would require an experimental dataset with higher precision.

We also investigated whether the anomalous behavior for SARS-2 described above could be responsible for the larger difference between the correlations computed over the training vs. test sets than was observed for influenza (compare panel C of Figure 4 with panel C of Figure 5), i.e., overfitting to the training set. To achieve this, we found the 84 = 70 of the 95 = 126 training/test partitions that contain the SARS-2 strain in the training set and recomputed the average in the correlations using only those strains. Figure 5C (blue symbols; see figure caption) shows that including the SARS-2 titers in the training data slightly lowered $c_{P}$ for the training sets and increased it for the test set, which indicates reduced overfitting to training data. We interpret this finding as an additional indication that the IgG titers measured against the SARS-2 strain behave differently from the other titers (e.g., that the sequence similarity is not the main titer determinant). It can also be interpreted as underscoring the importance of a diverse training set in reducing overfitting.

Finally, in Figure 5D, we colored the structure of the SARS-2 RBD by residue weights from the model; the weights are also plotted as a function of residue position in Figure S4B. The residues that make the highest contributions to the antibody binding are not at the tip of the RBD, which contacts the angiotensin (ACE2) receptor prior to target cell entry, but near the middle region. According to the X-ray and Cryo-EM structures of Barnes et al. [42], this part of the RBD contains epitopes which bind class 3 neutralizing antibodies, such as Regeneron REGN10987 [43] bnAb. Thus, the weights suggest that the vaccines elicit antibodies that target the more conserved parts of the RBD, in accord with the vaccine design [21]. However, as is the case for influenza, epitope mapping experiments using, e.g., competitive binding or mutational analysis, would be needed to test the epitope predictions.

### 3.3. mRNA Influenza Vaccine

**Figure 6:**
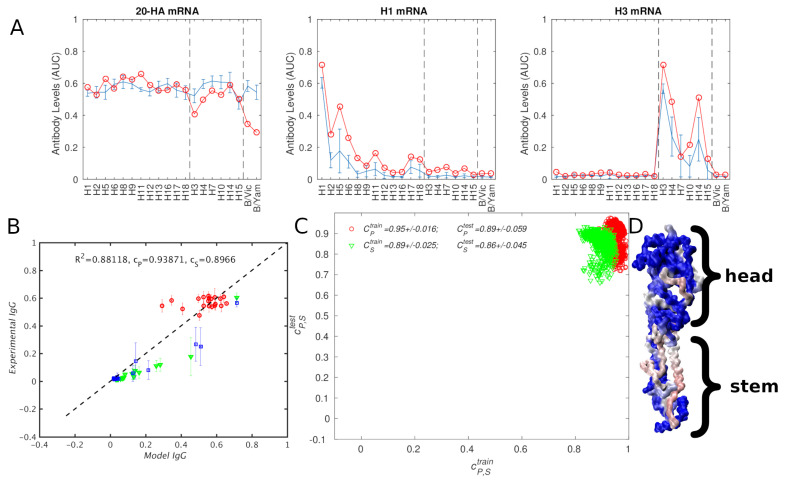
Comparison of model results with the experimental IgG titers for mRNA influenza vaccination [44]. (**A**,**B**): Best fit to experiment; in (**A**), the subplot title indicates the vaccine, the titers are shown for the same 20 antigens (see Table S1). The experimental titers are shown with error bars in blue, and the model results are shown as red circles. The vertical dashed lines separate HA group 1, HA group 2, and HB antigens (going from left to right); in (**B**), red circles, green triangles, and blue squares correspond to 20-HA, H1, and H3 vaccinations, and to the panels in A (left to right). (**C**): Comparison of 1000 training/testing splits in which 10 strains were randomly chosen for fitting (training) and the remaining 10 for testing (red ∘: $C_{P}$, green ▿: $C_{S}$). (**D**): Influenza hemagglutinin (PDB ID: 3LZG [39]) monomer colored by model weights using the color map blue-gray-red (corresponding to low-medium-high).

Although we developed our "average-strain" model to interpret vaccination data obtained from mosaic nanoparticle vaccines, which are hypothesized to improve elicitation of bnAbs via antibody bivalency [18,19,21,28], it is also useful to test the applicability of the model to other vaccine formulations. Further, Ives et al. [17] that the adaptive response can be targeted toward conserved immunosubdominant HA epitopes by manipulating antigen concentration in a cocktail of soluble HA ectodomains. Thus, we reparametrized the model using the mRNA vaccination data of Arevalo et al. [44], who vaccinated mice with a cocktail of mRNA lipid nanopartices encoding 20 different HAs corresponding to all known influenza subtypes. As controls, the authors also vaccinated mice with mRNA containing only H1 or H3 hemagglutinins. The model results are compared with the experiment in Figure 6, and summarized in Table 6. The strains composing the vaccines are listed in Table S1. The best model fit to the experiment, which uses all available titers, had an rmse of 0.09, twice the rms experimental error bar of 0.045. Qualitatively, it can be seen from Figure 6A that some of the model values are outside the experimental error bar. The difference was particularly large for the 20 HA vaccine titers against the two type B HA strains (Figure 6A, left panel). This is not very surprising, because the vaccination cocktail contained 18 type A strains, but only 2 B strains. Therefore, the "average" strain in the model was heavily biased toward type A and away from type B, which in part explains the low titers. Further, the model appeared to "compensate" for the low B titers by somewhat overestimating titers to type A HAs, which was especially visible in Figure 6A (middle panel). Given that the experimental titers against type B HAs in the 20 HA sera were generally as high as those for the type A strains, the discrepancy suggests that the average strain model assumed here may not be appropriate for cocktails composed of very dissimilar strains, such as those from type A and B hemagglutinins. As a test, we recomputed the best fit to experimental IgGs without any of the experimental titers against the B strains, and found the abovementioned overestimation of titers against type A strains to be somewhat reduced (see Figure S3). This appears to confirm that the discrepancy was caused, in part, by modeling a cocktail of antigens that were too dissimilar. Notably, this was not a rigorous test, because, in the refitting, we simply ignored data for the two B antigens, which were present in the 20 HA vaccine that elicited the actual post-vaccination sera, i.e., we do not know how the model would perform if the cocktail did not contain the B antigens. Although we currently do not have a quantitative approach to determine the minimum appropriate similarity between different strains in a cocktail, on the basis of pairwise sequence alignment scores computed for the antigenic cocktails considered here, we suggest a minimum cutoff of 30% (using BLOSUM50 scoring). This suggestion arises from the observation that all pairwise similarities computed here are above 35%, except those involving an influenza type B strain and and a type A strain, which are at or below 20% (see Figures S10–S12).

Despite the abovementioned errors, the model values generally correlate well with the experiments, producing $c_{P}$ and $c_{S}$ coefficients at or above ∼0.9 (Figure 6A–C). A notable exception to this observation can be seen in Table 6 section II, in which we split the data into training/test sets along vaccine definitions. Specifically, we trained the model using two of the three available vaccine titer sets, and used the remaining one for testing. If the training test only includes vaccines H1 and H3, the test on the remaining (20 HA) dataset produces no correlation to experiment (see Figure S7). However, this should be a surprise, because good models can only be expected to recapitulate data that are well represented in the training set.

Finally, in Figure 6D, we colored the structure of an HA spike using the residue weights determined from the model fit. It can be seen that the weights are significant for the stem region, as well as for the head, qualitatively, but not quantitatively, similar to the nanoparticle vaccine case. A comparison between the np-vaccine and mRNA-vaccine weights is shown in Figure S4. Overall, the np-vaccine weights seemed to be more concentrated in fewer regions/epitopes, whereas the mRNA-vaccine weights were more spread out, perhaps suggesting a more heterogeneous population of Abs.

### 3.4. Application to Vaccine Efficacy Prediction

**Figure 7:**
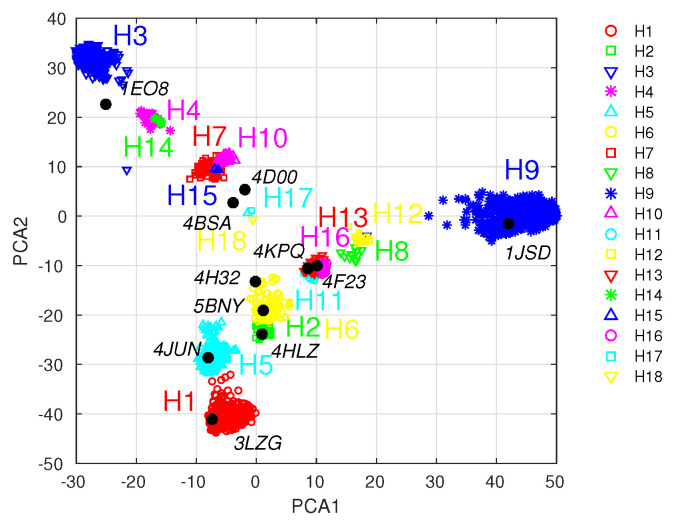
Two–dimensional projections of influenza hemagglutinin sequences onto principal components. Colors: projections of avian, swine, and human influenza type A spike protein sequences spanning the years 1918–2019 and subtypes 1–18, which were downloaded from the NIH influenza research database [38]; the sequences were clustered to 97% identity. Principal component analysis (PCA) was performed in MATLAB [35], as described in *Methods*; black bullets: projections of 11 antigens with solved Xray crystal structures (labeled with PDB codes). The strains corresponding to the PDB IDs and their sequence accession numbers are listed in Table S2.

**Figure 8:**
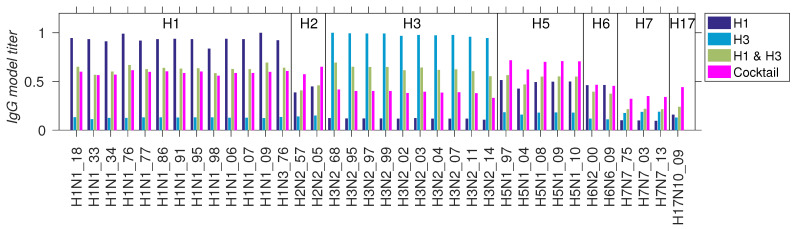
Comparison of predicted IgG titers for four hypothetical np vaccines using data of Cohen et al. [20]. *Cocktail* refers to the mixture of 11 HA antigens, as given in Table S2.

Our final result is an example application of the model to a comparison of several hypothetical vaccines with different antigen compositions. For model training, we used the np influenza data [20] described above in Section 3.1, which provide the model parameters α and β (see also Table 1, top row), and residue weights W. We chose four np vaccine compositions, which are given in Table S2, H1 homotypic, H3 homotypic, H1 and H3 mosaic, and 11 HA mosaic. The strains for the 11 HA mosaic vaccine were chosen to provide broad antigenic coverage within the influenza landscape, illustrated using principal component analysis (PCA) in Figure 7. For the convenience of a future experimental investigation, the chosen HA sequences all have available crystal structures, which would facilitate structural analysis or antigen engineering. These strains correspond to the set $\hat{V}$ in Section 2.3, and are listed in Table S2. For the test set ($T\in \hat{T}$), we chose a 36-antigen panel of Ives et al. [17], spanning multiple influenza subtypes over the years 1918–2014 (Table S2). The vaccination and test sets were added to the original MSA ($\hat{X}$) for the model, which associates the model residue weights with the new sequences. The model IgG titers were computed from Equations (2)–(4), and the results are compared in Figure 8. The figure illustrates several expected outcomes, e.g., that the H1 homotypic vaccine does not elicit significant Ab titers against group II HAs H3 and H7. Likewise, the H3 homotypic vaccine elicits predominantly anti-H3 titers. The 11 HA cocktail vaccine appears to elicit the greatest breadth, but at the expense of a lower anti-H3 response, relative to the H1 and H3 combination. These model predictions would benefit from future experimental validation. Since the predictions were generated with the np influenza parametrization, the experimental validation procedure should follow the mouse vaccination protocol of Cohen et al. [20]. Briefly, four groups of 4–6-week-old female Balb/c mice, with four or more mice per group, could be vaccinated with the nanoparticle vaccines proposed above; two additional groups could serve as positive (e.g., seasonal quadrivalent vaccine) and negative (e.g., PBS solution) controls. The mice would be primed on day 0 and boosted on day 14 via intraperitoneal injections of 20 μg of antigen in 200 μL of 50% *v*/*v* of Sigma Adjuvant System. ELISA assays would be carried out on sera collected on day 21, using the antigen test panel in Table S2, and the determined area-under-curve (AUC) signals correlated with the present predictions.

## 4. Concluding Discussion

Viral escape poses a major challenge for the development of vaccines for highly mutable pathogens such as HIV, influenza, and coronavirus, because rapidly emerging pathogen variants render host immunity raised by administered vaccines obsolete. Thus, it is important to design vaccines that elicit broadly reactive sera able to counteract a wide variety of pathogen mutants, especially those not present in the vaccines. Toward this goal, we parametrized a model of reactivity of the sera obtained from vaccinated naïve mice with influenza or coronavirus nanoparticle vaccines [20,21]. The model was able to recapitulate the experimental Ab titers to within experimental uncertainty, and produced Pearson and Spearman correlation coefficients in the range 0.6–0.9, depending on the dataset used. Notably, the model parametrization procedure required only a modest amount of data (e.g., 20 titer values used for parametrization/training and 16–20 for testing). However, additional experimental data would improve model quality, e.g., by reducing experimental uncertainty in the average titers (using more test subjects), by incorporating more strains into vaccine cocktails or testing panels, or by allowing the use of more complex models with additional parameters, such as multiple residue weights to represent different epitopes. In principle, sophisticated neural networks could be trained to model vaccine efficacy, such as those used to predict properties of small molecules [45,46], or antibody structures [47]. However, such models typically require very large amounts of data (thousands to millions of data points) to achieve high accuracy. Such data may be costly, even impossible, to obtain in vaccination experiments and would probably not be transferable between vaccine types or vaccination protocols (a limitation also shared by our approach). Although the present model yields good results with few data points, using it required significant human input, e.g., choice of the functional form of the similarity function (Equation (4)) or amino acid encoding. For example, using the Atchley et al. [32] encoding for the influenza vaccines yields a somewhat lower quality fit to the vaccine titers, with several model points outside of the experimental error bar (see Supplementary Figure S1B), and the distribution of residue weights was high for a portion of the hemagglutinin stem that is expected not to be exposed to antibodies in a trimer (Figure S1D). For these reasons, and with the additional justification that the Grantham [31] encoding appears to be more grounded in the physical properties of amino acids, we chose the Grantham encoding, rather than the more empirical fit of Atchley et al. [32].

The present model was developed to interpret vaccination data obtained from mosaic nanoparticle vaccines. The main reason for this choice is that such vaccines have been shown to direct the immune response to more conserved immunosubdominant epitopes [18,19,21,28], which are associated with eliciting broadly neutralizing antibodies (bnAbs) [40,48]. The underlying reason for the greater breadth associated with such vaccines appears to be antibody avidity (see Figure 3C). Specifically, because antibodies and their membrane-bound counterparts—BCRs—have two fragments for antigen-binding (Fabs), and because antigen binding translates into a proliferation signal for the corresponding B cells during antibody evolution [49], mosaic nanoparticle vaccines are expected to elicit antibodies directed to more conserved parts on the antigen [18].

Since the model assumes that the maturing antibodies see an “average” strain, the encouraging results obtained here suggest that the vaccination sera contain bnAbs that are cross-reactive with multiple antigen strains. However, to conclude this with confidence would likely require evaluating fits to alternative models (see, e.g., the Supporting Material), and showing that the present model produces superior results. This may not be possible with the presently available experimental datasets, which contain large statistical errors.

The ability to predict vaccine efficacy against an arbitrary pathogenic variant is clearly useful. However, because the space of possible amino acid mutations is astronomical (even if not all 20 natural amino acids are allowed at all sequence positions), it is generally not possible to predict which specific strains will emerge in the future. Nevertheless, the effects of viral mutations can be partially characterized by high-throughput mutagenesis experiments [7] to construct viral fitness landscapes, from which the most fit mutant sequences can be tested using models such as those presented here. Alternatively/additionally, one could use such models to maximize antibody breadth over a space of candidate vaccine designs (see e.g., Ref. [50]) relative to an established panel of antigen variants [51].

Although the results described here are encouraging, it is important to discuss the limitations of the modeling approach. First, the model parametrizations described here are very unlikely to be directly transferable between different vaccine formulations because of a multitude of complex factors that determine the overall immune response, which are not explicitly represented in the models. Examples of such factors are nanoparticle vs. soluble ectodomain presentation, route of administration, choice of adjuvant, or choice of boosting protocol. This can already be seen here by comparing the model parameters obtained from fitting the model to nanoparticle vs. mRNA vaccination data (Figure 4 and Figure 6, respectively). We expect the models to be somewhat general only within a particular class of vaccine formulation, i.e., after the factors such as those mentioned above are decided upon and fixed, so that the remaining differences between vaccines are dominated by antigen sequence variability. Even in these restricted scenarios, the models will likely need to be reparametrized. Fortunately, this is a straightforward task, given the simplicity of Equations (2)–(4), and is not expected to require excessive experimentation. Further, if sufficient data are available for training, model sophistication can readily be improved. For example, an assumption that is made in our modeling is that a single weight array W can adequately describe the binding between the antibodies and the antigens. This assumption implies that all of the modeled vaccines are sufficiently similar so as to elicit antibodies to the same antigen epitopes, which is not true, in general. Thus, a simple generalization of the present model is to include multiple weight arrays, which can be achieved, e.g., by replacing overall similarity function by a sum of constituent functions, e.g., for two weight arrays, $W_{1}$ and $W_{2}$,

(13) $$f_{total}=f\left(d_{1};\alpha_{1},\beta_{1}\right)+f\left(d_{2};\alpha_{2},\beta_{2}\right),$$

where $d_{i}$ is as in Equation (3), with $W_{i}$ in place of W. The fitting/training of such models would be very similar to the procedure described here. Although an advantage of the present modeling is that structural information about neither the antigens nor the antibodies is required, recent advances in structure prediction methods, e.g., AlphaFold3 [52], RoseTTAFold [53], ProtTrans [54], or ESM-2 [55], could be used to predict antigen structures in solution or in complex with antibodies. Such structures could provide information on epitope accessibility or immunogenicity, which could be incorporated into a future version of the model via additional or alternative epitope weights.

Finally, we do not explicitly take into account any preexisting immunity, or original antigenic sin (OAS), which is known to skew antibody responses toward previously encountered strains [56]. In principle, OAS could be incorporated in the model by including pre-vaccination titers to determine the direction of the skewness in antigen-experienced subjects.

## Figures and Tables

**Table 1:** Values of model parameters defined in *Methods*.

	α	β	τ	*D*	$n_{\max}^{†}$
Influenza-np	1	4	0.015–0.03	0.49	3000
Coronavirus-np	1	5	0.02–0.04	0.3	3000
Influenza-mRNA	1.4	9	0.01	0.2	4000

^†^ Number of gradient descent iterations in Equation (6).

**Table 2:** Influenza strains used in the experiments [20] and in the present modeling. V1, V2, V4, and V8 refer to the vaccination cocktails.

ABBREVIATION	STRAIN NAME	ACCESSION CODE	V1	V2	V4	V8
C09	A/California/04/2009(H1N1)	ACS45035	✓	✓	✓	✓
V04	A/Viet Nam/1203/2004	AAW80717.1	–	–	✓	✓
J57	A/Japan/305/1957(H2N2)	AAA43185.1	–	–	–	✓
HK99	A/guinea fowl/Hong Kong/WF10/99	AAO46082.1	–	–	–	✓
AI68	A/Aichi/2/1968	AAA43178.1	–	✓	✓	✓
SH13	A/Shanghai/1/2013	EPI439486	–	–	✓	✓
JX13	A/Jiangxi/IPB13/2013(H10N8)	AHK10761	–	–	–	✓
HB09	A/swine/HuBei/06/2009(H4N1)	AFV33926	–	–	–	✓
P10	A/flat-faced bat/Peru/033/2010	AGX84934.1	–	–	–	–
WA79	A/wedge-tailed shearwater/Western Australia/2576/1979	ABB88138.1	–	–	–	–

**Table 3:** Coronavirus strains used in the experiments [21] and in the present modeling.

NAME	ACCESSION CODE	HOST	V1	V4A	V4B	V8
SARS-2	MN985325.1	human	✓	✓	–	✓
RaTG13	QHR63300	bat	–	✓	–	✓
SHC014	KC881005	bat	–	✓	–	✓
Rs4081	KY417143	bat	–	✓	–	✓
Pang17	QIA48632	pangolin	–	–	✓	✓
RmYN02	EPI_ISL_412977	bat	–	–	✓	✓
Rf1	DQ412042	bat	–	–	✓	✓
WIV1	KF367457	bat	–	–	✓	✓
SARS	AAP13441.1	human	–	–	–	–
Yun11	JX993988	bat	–	–	–	–
BM-4831	NC_014470	bat	–	–	–	–
BtKY72	KY352407	bat	–	–	–	–

V1, V4a, V4b, and V8 refer to the vaccination cocktails.

**Table 4:** Summary of model performance using influenza nanoparticle vaccination data.

**Enc-Type**	$c_{P}^{trn}$ (m ± sd)	$c_{P}^{tst}$ (m ± sd)	$c_{S}^{trn}$ (m ± sd)	$c_{S}^{tst}$ (m ± sd)	$p_{val}^{c_{P},tst}$ (min/max/avg)	$c_{P}^{hld}\left(p_{val}\right)$	$c_{S}^{hld}\left(p_{val}\right)$
I. Train/test split by antigen							
Gr-mosaic	0.91 ± 0.03	0.84 ± 0.05	0.92 ± 0.03	0.85 ± 0.05	$2.9\times 10^{-9}/3.1\times 10^{-3}/4.8\times 10^{-5}$	N/A	N/A
At-mosaic	0.91 ± 0.03	0.74 ± 0.13	0.91 ± 0.03	0.78 ± 0.09	$4.4\times 10^{-9}/8.4\times 10^{-2}/6.7\times 10^{-3}$	N/A	N/A
Gr-np-mix	0.77 ± 0.06	0.66 ± 0.11	0.73 ± 0.07	0.65 ± 0.15	$3.1\times 10^{-6}/1.3\times 10^{-1}/9.3\times 10^{-3}$	N/A	N/A
II. Train/test split by vaccine							
Gr-mosaic	0.92 ± 0.04	0.84 ± 0.10	0.92 ± 0.03	0.86 ± 0.07	$3.4\times 10^{-9}/2.0\times 10^{-3}/3.4\times 10^{-4}$	N/A	N/A
At-mosaic	0.93 ± 0.03	0.77 ± 0.11	0.90 ± 0.03	0.78 ± 0.11	$9.7\times 10^{-7}/3.6\times 10^{-3}/1.0\times 10^{-3}$	N/A	N/A
Gr-np-mix	0.85 ± 0.20	0.71 ± 0.19	0.78 ± 0.19	0.70 ± 0.19	$1.8\times 10^{-10}/9.6\times 10^{-2}/1.6\times 10^{-2}$	N/A	N/A
III. With a “holdout” set (V8)							
Gr-mosaic	0.92 ± 0.02	0.74 ± 0.07	0.92 ± 0.04	0.70 ± 0.09	$1.6\times 10^{-3}/1.2\times 10^{-1}/1.9\times 10^{-2}$	0.79 ($6.9\times 10^{-3}$)	0.73 ($2.1\times 10^{-2}$)
At-mosaic	0.92 ± 0.02	0.71 ± 0.11	0.88 ± 0.05	0.69 ± 0.13	$1.4\times 10^{-4}/7.7\times 10^{-1}/3.7\times 10^{-2}$	0.77 ($9.4\times 10^{-3}$)	0.71 ($2.8\times 10^{-2}$)
Gr-np-mix	0.90 ± 0.04	0.62 ± 0.09	0.66 ± 0.12	0.53 ± 0.14	$9.8\times 10^{-3}/3.4\times 10^{-1}/7.0\times 10^{-2}$	0.70 ($2.5\times 10^{-2}$)	0.63 ($5.3\times 10^{-2}$)

Abbreviations **Gr** and **At** refer to the Grantham [31] and Atchley [32] encodings (**Enc**), respectively; **type** refers to the nanoparticle type which can be mosaic or np-mix (see Figure 3B) [20]; subscripts *trn* and *tst* refer to training and test sets, respectively. *p*-values ($p_{val}$) are reported for the Pearson correlation coefficient computed over the test set as triplets, indicating the minimum, maximum, and average values. For testing on the holdout set, the performance of the models is indicated by Pearson ($c_{P}^{hld}$) and Spearman ($c_{S}^{hld}$) correlation coefficients with the corresponding *p*-values in parentheses; N/A indicates that a holdout set was not chosen.

**Table 5:** Summary of model performance using coronavirus vaccination data. The quantities listed are the same as those described for influenza in Table 4. The model was used with the Grantham encoding.

$c_{P}^{trn}$ (m ± sd)	$c_{P}^{tst}$ (m ± sd)	$c_{S}^{trn}$ (m ± sd)	$c_{S}^{tst}$ (m ± sd)	$p_{val}^{c_{P},tst}$ (min/max/avg)	$c_{P}^{hld}\left(p_{val}\right)$	$c_{S}^{hld}\left(p_{val}\right)$
I. Train/test split by antigen						
0.86 ± 0.04	0.59 ± 0.13	0.84 ± 0.06	0.56 ± 0.13	$9.6\times 10^{-5}/2.5\times 10^{-1}/3.9\times 10^{-2}$	N/A	N/A
II. Train/test split by vaccine						
0.89 ± 0.03	0.65 ± 0.16	0.84 ± 0.12	0.64 ± 0.16	$1.8\times 10^{-6}/3.8\times 10^{-2}/1.5\times 10^{-2}$	N/A	N/A
III. With a “holdout” set (V8)						
0.87 ± 0.04	0.68 ± 0.12	0.86 ± 0.04	0.63 ± 0.15	$9.1\times 10^{-4}/6.8\times 10^{-1}/6.5\times 10^{-2}$	0.80 ($9.0\times 10^{-3}$)	0.83 ($8.3\times 10^{-3}$)

**Table 6:** Summary of model performance using influenza mRNA vaccination data [44]. The quantities listed are the same as those described for nanoparticle influenza in Table 4. The model was used with the Grantham encoding. For the train/test split by antigen, 1000 antigen panels were generated, in which 10 out of 20 antigens were chosen for training; the remaining antigens were used for testing. For the train/test split by the vaccine, we used data for two out of three vaccine titer sets for training, and the remaining one, for testing.

$c_{P}^{trn}$ (m ± sd)	$c_{P}^{tst}$ (m ± sd)	$c_{S}^{trn}$ (m ± sd)	$c_{S}^{tst}$ (m ± sd)	$p_{val}^{c_{P},tst}$ (min/max/avg)
I. Train/test split by antigen				
0.95 ± 0.02	0.89 ± 0.06	0.89 ± 0.03	0.86 ± 0.05	$1.0\times 10^{-24}/5.8\times 10^{-11}/2.6\times 10^{-13}$
II. Train/test split by vaccine				
0.96 ± 0.03	0.58 ± 0.5	0.83 ± 0.05	0.52 ± 0.5	$3.7\times 10^{-30}/5.7\times 10^{-17}/1.9\times 10^{-17}$

## Data Availability

The computer code used for the model in this paper, including the experimental dataset used for training, and partial results, can be found online at https://github.com/ovchinnv/npvaccine-titer-model2022.git, accessed on 29 November 2024.

## Supplementary Materials

The following supporting information can be downloaded at https://www.mdpi.com/article/10.3390/antib14010006/s1. Supporting text discussing alternative models, sequence alignments of antigenic sequences, Figures S1–S12 and Tables S1 and S2 accompany this article.
