# Supplementary material for: Phenomenological Modeling of Antibody Response from Vaccine Strain Composition
Source: Antibodies (Basel). 2025 Jan 16;14(1):6. doi: 10.3390/antib14010006 (PMC11755667; doi:10.3390/antib14010006)
Supplement: Supplementary file 1 [file antibodies-14-00006-s001.zip › antibodies-3372899-supplementary.pdf]

Supporting Text

Alternative IgG models

In this study, we hypothesized that, after a cocktail vaccination, maturing B-cells develop affinity to an "average" epitope structure, which is why the affinity of the elicited response to any antigen strain was determined by the distance between a test strain ($\boldsymbol{T}$) and the average vaccine strain $\overline{\boldsymbol{V}}$. While the satisfactory agreement between our modeling results and experiment appears to support this hypothesis, other possibilities can also be considered.

For example, in an alternative maturation scenario, any particular immature BCR would have significant affinity only to a single antigen in the cocktail. In this case, the selection forces driving the AM of the BCR will be dominated by that antigen. If most of the Abs elicited by vaccination evolved in this way, we would expect the Ab repertoire to be dominated by an ensemble of strain-specific Abs; in this case, we could assume that the affinity of the elicited response to an antigen strain $\boldsymbol{T}$ will be a function of the average distance from $\boldsymbol{T}$ to the constituent vaccine strains. Specifically, let $\widehat{\boldsymbol{V}}$ represent a vaccine cocktail, as in the main text. For a sequence $\boldsymbol{T} \in \widehat{\boldsymbol{X}}$, the model IgG titer elicited by $\widehat{\boldsymbol{V}}$ could be

$$\begin{aligned} IgG^M(\widehat{\boldsymbol{V}}, \boldsymbol{T}) &\equiv f\left\{\frac{1}{v}\sum_{\boldsymbol{V}\in\widehat{\boldsymbol{V}}} d(\boldsymbol{V}, \boldsymbol{T}; \boldsymbol{W})\right\} \\ &= f\left\{\bar{d}(\widehat{\boldsymbol{V}}, \boldsymbol{T})\right\}, \end{aligned} \tag{S14}$$

where $\bar{d}(\widehat{\boldsymbol{V}}, \boldsymbol{T})$ is the average distance between the vaccine cocktail $\widehat{\boldsymbol{V}}$ and strain $\boldsymbol{T}$. One could use the same functional form for the similarity function f (Eq. (4)) for simplicity, or consider more complicated ones. The weights and similarity function coefficients could be optimized by fitting to experimental IgG measurements, as described in the main text.

While Eq. (S14) assumes that IgG titers are determined by the average distance between the test strain and each strain in the vaccine cocktail, alternatively, one could also average the similarities directly, *i.e.*,

$$\begin{aligned} IgG^M(\widehat{\boldsymbol{V}}, \boldsymbol{T}) &\equiv \frac{1}{v}\left\{\sum_{\boldsymbol{V}\in\widehat{\boldsymbol{V}}} f(d(\boldsymbol{V}, \boldsymbol{T}; \boldsymbol{W}))\right\} \\ &= \bar{f}(d(\widehat{\boldsymbol{V}}, \boldsymbol{T})), \end{aligned} \tag{S15}$$

Discriminating between such models reliably would almost certainly require significant reductions in statistical errors, compared to the data sets used here. We hope that future vaccination data sets will permit such analysis.

Supporting Figures

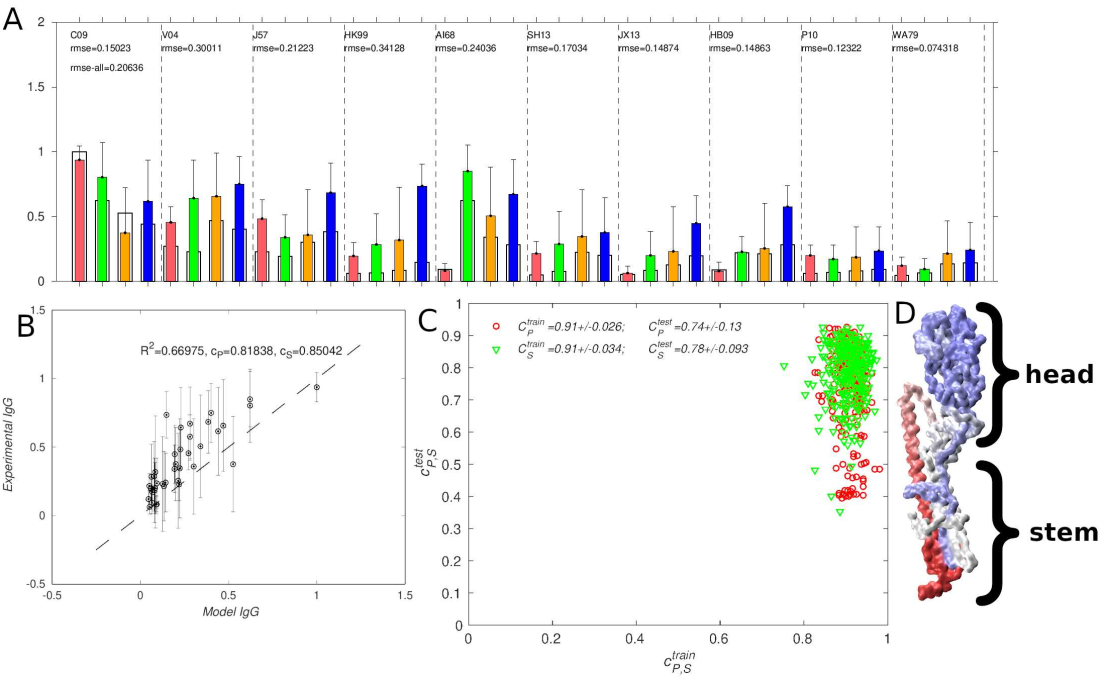

Figure S1. Comparison of model results using the Atchley et al. [32] amino acid encoding with the experimental IgG titers for influenza[20]; A & B: best fit to experiment; in A, the colors correspond to experimental data for vaccines in Table 2 (red ■ V1, green ■ V2, orange ■ V4, blue ■ V8), and the outer transparent bars are model values; C: comparison of 252 possible fits in which 5 strains were used for fitting (training) and 5 for testing (red ○ : C_P, green ▽ : C_S) ; D: influenza hemagglutinin (PDB ID: 3LZG[39]) monomer colored by model weights using the colormap blue-gray-red (corresponding to low-medium-high). C_P and C_S are the Pearson and Spearman rank correlation coefficients, respectively; *rmse* denotes root-mean-squared error relative to the experiment.

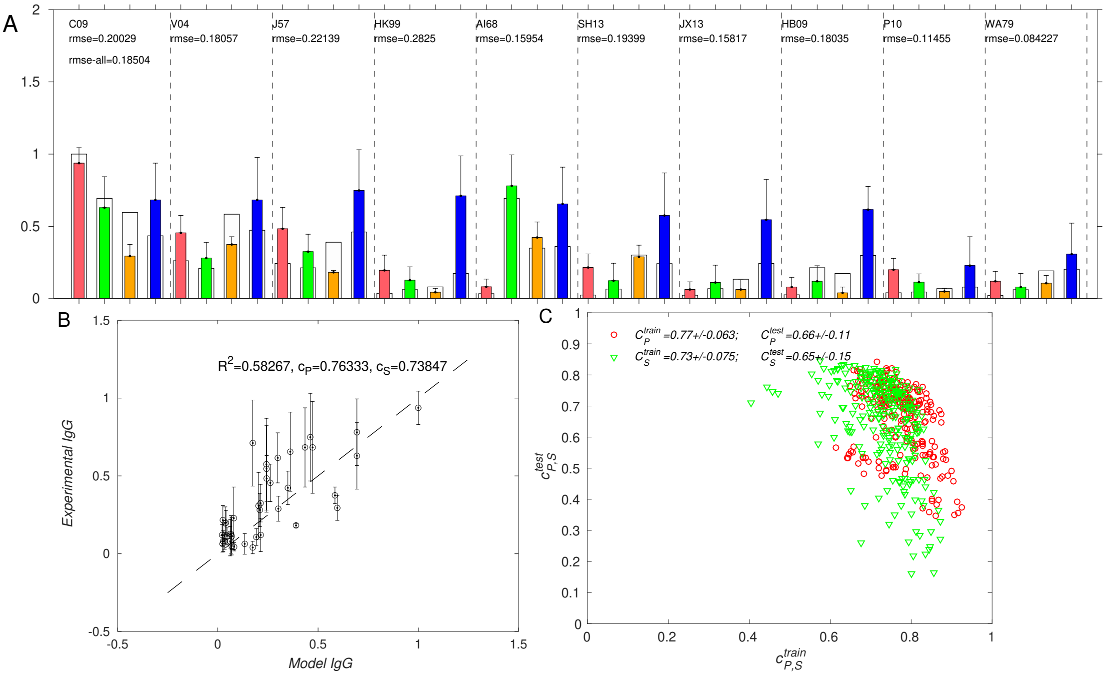

Figure S2. Comparison of model results with the experimental IgG titers for influenza[20] (np-mix formulation); A & B: best fit to experiment; in A, the colors correspond to experimental data for vaccines in Table 2 (red ■ V1, green ■ V2, orange ■ V4, blue ■ V8), and the outer transparent bars are model values; C: comparison of 252 possible fits in which 5 strains were used for fitting (training) and 5 for testing (red ○ : C_P, green ▽ : C_S);

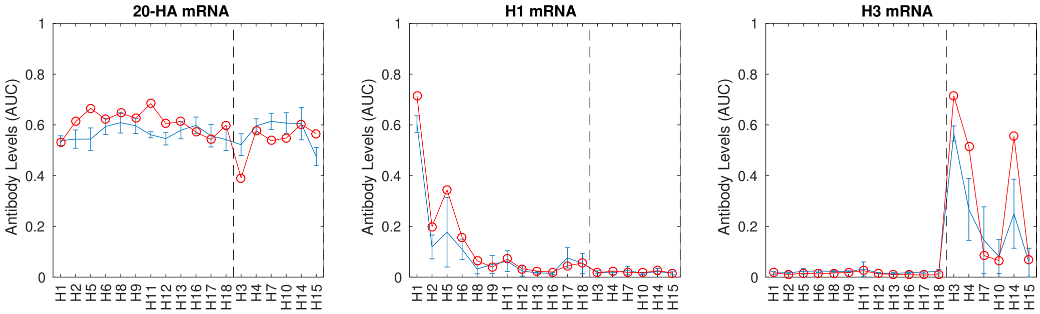

Figure S3. Comparison of model results with the experimental IgG titers for mRNA influenza vaccination.[44] The symbols are as described for figure 6A, except that the model was reparametrized without including titers against the two influenza B strains.

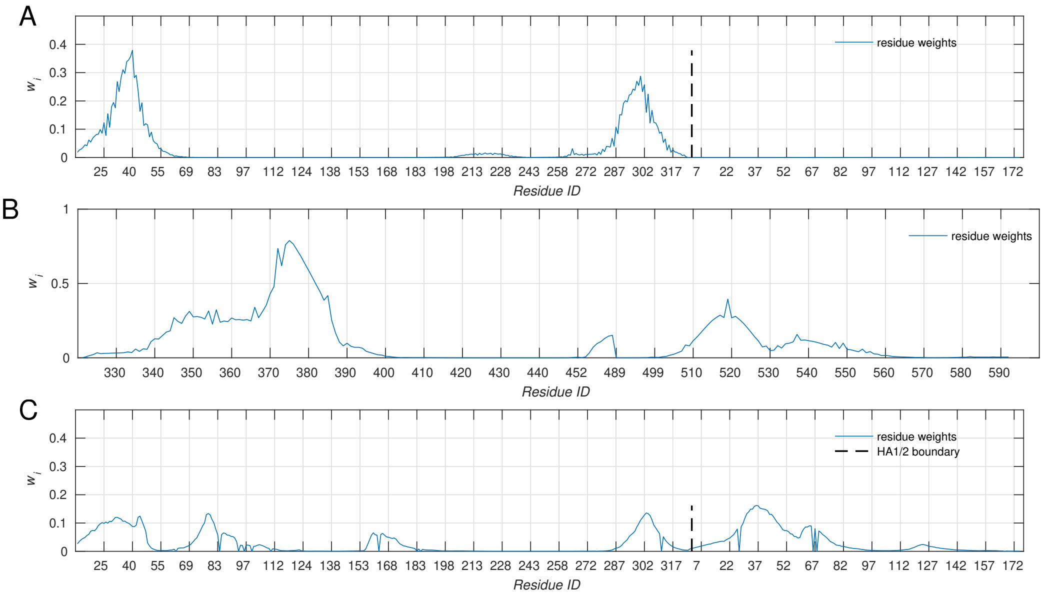

Figure S4. Optimized residue weights for np-influenza (A), coronavirus (B), and mRNA-influenza (C). The residue ID labels correspond to the residue numbering in the PDB files (3LZG and 6VXX for influenza and coronavirus, respectively). In (A) & (C), the vertical dashed line separates HA subunits 1 (left of line) and 2 (right of line).

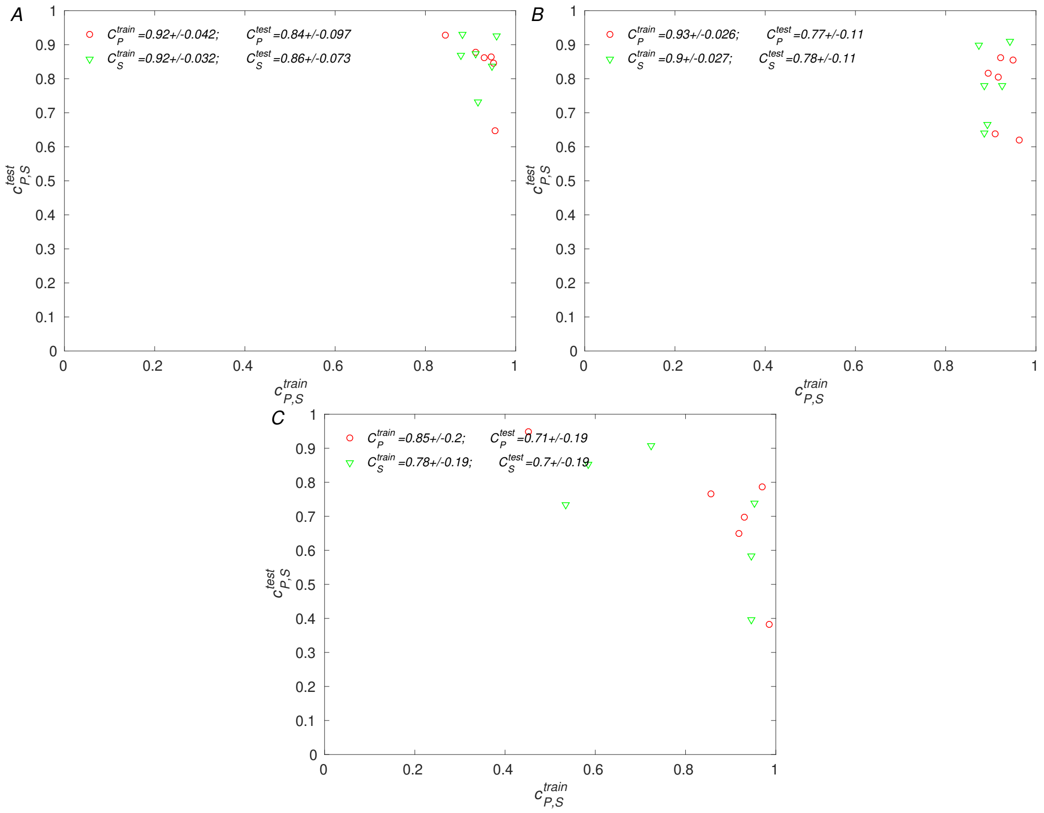

Figure S5. Comparison of model results with the experimental IgG titers for influenza np vaccine titers.[20] Training was performed with 50% of the experimental data set, choosing any two vaccine IgG titer sets, with the remaining two used for testing, which results in six train/test partitions (see main text). The panels correspond to the vaccines in Table 4 (section II) as follows; A: Gr-mosaic, B: At-mosaic, C: Gr-np-mix.

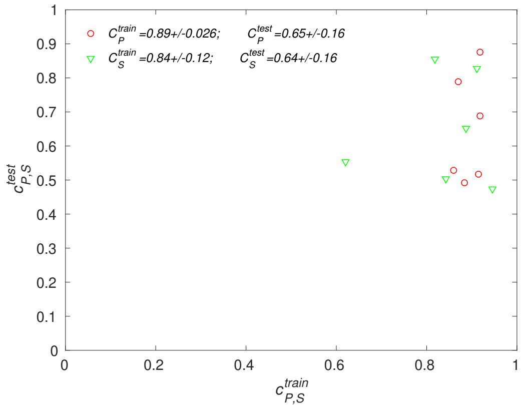

Figure S6. Comparison of model results with the experimental IgG titers for coronavirus.[21] The panels correspond to the vaccines in Table 5 (section II). The remaining details are the same as those for the influenza data in Fig. S5.

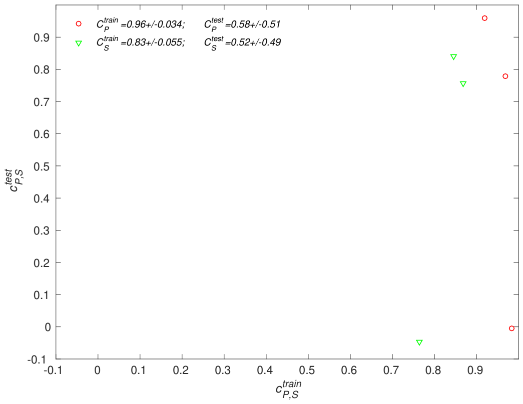

Figure S7. Comparison of model results with the experimental IgG titers for influenza mRNA vaccine titers.[44] The panels correspond to the vaccines in Table 6 (section II). All possible pairs of vaccine titer sets were chosen for training, with the remaining one used for testing, (three train/test partitions). In the partition without the 20-HA titer in the training data, the model has no predictive power (see main text).

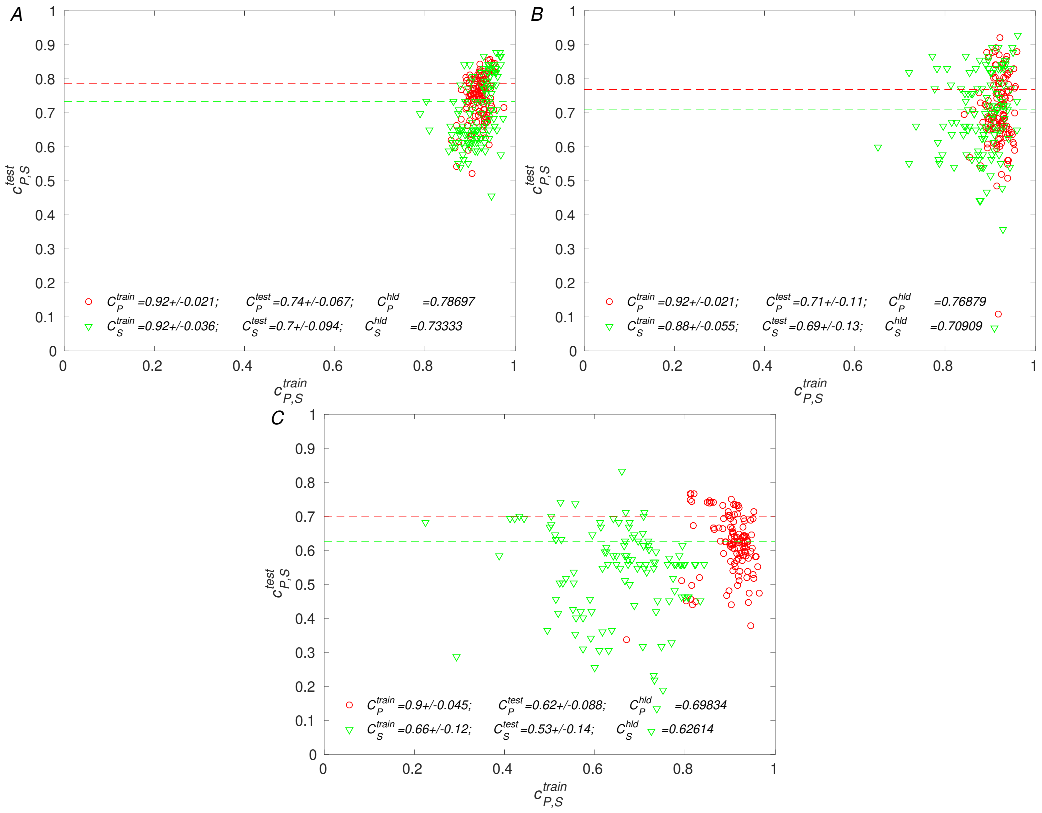

Figure S8. Comparison of model results with the experimental IgG titers for influenza.[20] Testing with a 'holdout' experimental data set, consisting of titers measured after vaccination with vaccine V8 (see main text). Training was performed with 70% of the remaining experimental data, which corresponds to ∼50% of the entire experimental data set. The scatter plots show the performance of the models that correspond to all possible samplings of the experimental data set. The dashed lines indicate Pearson and Spearman correlation coefficients for the holdout set. The panels correspond to the vaccines in Table 4 (section III) as follows; A: Gr-mosaic, B: At-mosaic, C: Gr-np-mix.

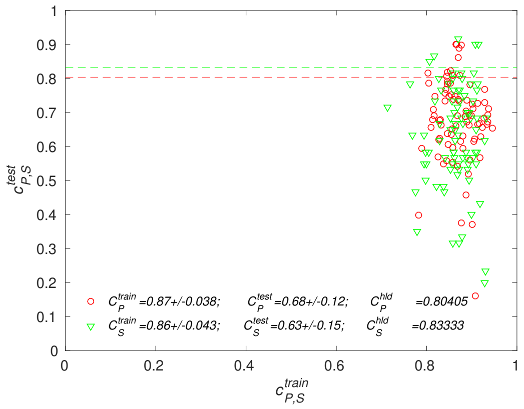

Figure S9. Comparison of model results with the experimental IgG titers for coronavirus.[21] Testing with a 'holdout' experimental data set, consisting of titers measured after vaccination with vaccine V8 (see main text). The remaining details are the same as those for the influenza data in Fig. S8.

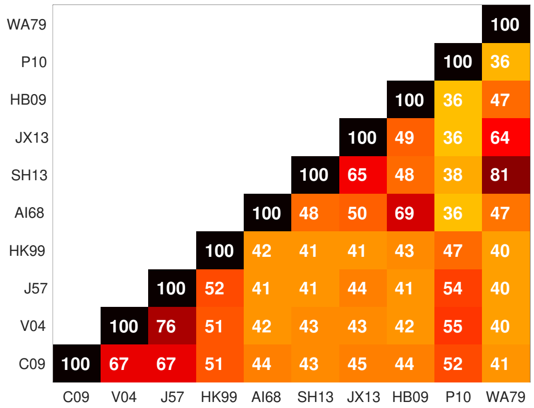

Figure S10. Pairwise similarity score matrix computed for the influenza hemagglutinin ectodomain sequences used in this study. Alignments were performed in Matlab using the Needleman-Wunsch algorithm, and scored with the block substitution matrix clustered at 50% similarity (BLOSUM50); scores were normalized to 100.

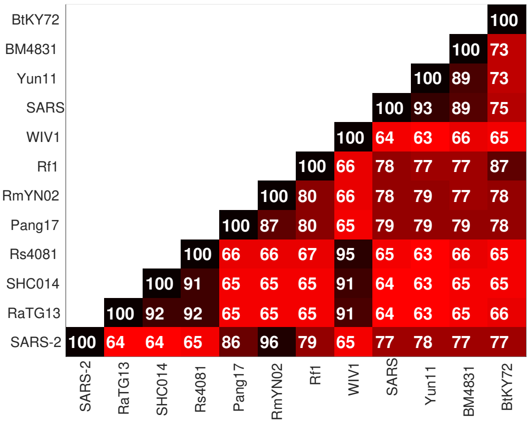

Figure S11. Pairwise similarity score matrix computed for the coronavirus RBD sequences used in this study. Alignments were performed as for influenza HA (see Fig. S10).

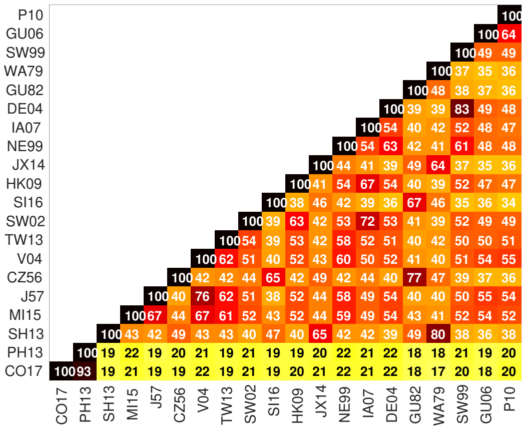

Figure S12. Pairwise similarity score matrix computed for the influenza hemagglutinin ectodomains used in the mRNA vaccine of Arevalo et al. [44] Alignments were performed as for Fig. S10.

Table S1. Influenza strains used in the experiments of Arevalo et al. [44]. H1, H3, and 20-HA refer to the vaccination cocktails.

STRAIN NAME	H1	H3	20-HA
A/Michigan/45/2015 (H1)	✓		✓
A/Japan/305/1957 (H2)			✓
A/duck/Czech/1956 (H4)			✓
A/Vietnam/1203/2004 (H5)			✓
A/Taiwan/2/2013 (H6)			✓
A/mallard/Sweden/24/2002 (H8)			✓
A/Singapore/INFIMH-16-0019/2016 (H3)		✓	✓
A/Shanghai/02/2013 (H7)			✓
A/Hong Kong/33982/2009 (H9)			✓
A/Jiangxi/09037/2014 (H10)			✓
A/shoveler/Netherlands/18/1999 (H11)			✓
A/mallard/Interior Alaska/7MP0167/2007 (H12)			✓
A/shorebird/Delaware/68/2004 (H13)			✓
A/mallard/Gurjev/263/1982 (H14)			✓
A/shearwater/West Australia/2576/1979 (H15)			✓
A/black headed gull/Sweden/5/1999 (H16)			✓
A/yellow shouldered bat/Guatemala/06/2010 (H17)			✓
A/bat/Peru/33/2010 (H18)			✓
B/Colorado/06/2017 (HBV)			✓
B/Phuket/3073/2013 (HBY)			✓

Supporting Data

Table S2. Influenza strains used in hypothetical vaccine efficacy test.

Strain Name	Accession #, PDB ID	H1	H3	H1 & H3	11-HA
Vaccine antigens					
A/Kagoshima/1/2009(H1N1)	ACT22035.1, 3LZG	✓		✓	✓
A/little yellow-shouldered bat/Guatemala/060/2010(H17)	AFC35438.1, 4H32				✓
A/Aichi/2/1968(H3)	AAA43178.1, 1EO8		✓	✓	✓
A/Japan/305/1957(H2N2)	ACV49600, 4HLZ				✓
A/Swine/Hong_Kong/9/98(H9)	EPI6943, 1JSD				✓
A/duck/Guangxi/2396/2004(H5N1)	ABC66534, 4JUN				✓
A/Hangzhou/1/2013(H7N9)	AGI60301, 4BSA				✓
A/gull/Maryland/704/1977(H13)	BAA14338.1, 4KPQ				✓
A/chicken/Germany/N/1949(H10N7)	ABI84534, 4D00				✓
A/chicken/Guangdong/S1312/2010(H6N2)	AHJ58072, 5BNY				✓
A/black-headed_gull/Sweden/2/99(H16)	AAV91214.1, 4F23				✓
Test antigen panel					
A/South_Carolina/1/18	AAD17229.1				
A/WSN/1933_TS61	ABF47955.1				
A/Puerto_Rico/8/1934(H1N1)	AFM71846.1				
A/New_Jersey/8/1976	ACQ99821.1				
A/USSR/90/1977(H1N1)	ABD95350				
A/Taiwan/01/1986(H1N1)	ABF21274				
A/Texas/36/1991(H1N1)	AAP34322				
A/Beijing/262/1995(H1N1)	AAP34323				
A/swine/Belgium/1/1998(H1N1)	ACN67524				
A/Solomon_Islands/3/2006	ABU99109.1				
A/Brisbane/59/2007(H1N1)	ACA28846				
A/California/04/2009(H1N1)	ACS45035				
A/duck/NZL/160/1976(H1N3)	ABB20429				
A/Guiyang/1/1957(H2N2)	ACD85231				
A/Canada/720/2005(H2N2)	AAY28987				
A/Hong_Kong/1/1968	AFG71887.1				
A/Nanchang/933/1995(H3N2)	AFG72625				
A/Sydney/5/1997(H3N2)	ACO95259				
A/Moscow/10/1999(H3N2)	AAT08002				
A/Fujian/411/2002(H3N2)	AFD64223				
A/Nymc_X-149C6(A/Puerto_Rico/8/1934-A/Wyoming/03/2003)_RX	ACF41889.1				
A/California/07/2004(H3N2)	ABW80975				
A/Brisbane/10/2007(H3N2)	ABW23353				
A/Victoria/361/2011(H3N2)	AGB08328				
A/Hong_Kong/4801/2014	EPI643118				
A/Hong_Kong/483/1997(H5N1)	ACZ48509.1				
A/Vietnam/1194/2004(H5N1)	ACR48873				
A/chicken/Vietnam/NCVD-016/2008(H5N1)	ACO07033				
A/Egypt/N05056/2009(H5N1)	ACT15357				
A/barn_swallow/Hong_Kong/1161/2010	AGC13463.1				
A/duck/Shantou/83/2000(H6N2)	ADG44842				
A/duck/Eastern_China/11/2009	EPI345637				
A/Kentucky/1a/1975	CY036871.1_ACL12085.1				
A/Netherlands/219/2003(H7N7)	AAR02640				
A/silkie_chicken/Shaoxing/5235/2013	AJJ91761.1				
A/little_yellow-shouldered_bat/Guatemala/153/2009	AFC35418.1				

```
Multiple Sequence Alignment of Influenza Hemagglutinin Ectodomains


C09             MKAI-LVVLLYTFATA--------NADTLCIGYHANNSTDTVDTVLEKNVTVTHSVNLLE  51
V04             MEK--IVLLFAIVSLV--------KSDQICIGYHANNSTEQVDTIMEKNVTVTHAQDILE  50
J57             MA---IIYLILLFTAV--------RGDQICIGYHANNSTEKVDTNLERNVTVTHAKDILE  49
HK99            METISLITILLVVTAS--------NADKICIGHQSTNSTETVDTLTETNVPVTHAKELLH  52
AI68            MKTIIALSYIFCLALGQDLPGNDNSTATLCLGHHAVPNGTLVKTITDDQIEVTNATELVQ  60
SH13            MNTQILVFALIAIIPT--------NADKICLGHHAVSNGTKVNTLTERGVEVVNATETVE  52
JX13            MYKIVVIIALLGAVK---------GLDKICLGHHAVANGTIVKTLTNEQEEVTNATETVE  51
HB09            MLSIVILFLLVAENSSQNYTGN----PVICMGHHAVANGTMVKTLTDDQVEVVAAQELVE  56
P10             MIT----ILILVLPIV--------VGDQICIGYHSNNSTQTVNTLLESNVPVTSSHSILE  48
WA79            MNTQIIVILVLGLSMV--------KSDKICLGHHAVANGTKVNTLTERGVEVVNATETVE  52
                *        .                  :*:*:::  .   *.*  :    *. : . :.

C09             DKHNGKLCKLRGVAPLHLGKCNIAGWILGNPECESLSTASSWSYIVETPSSDNGTCYPGD 111
V04             KKHNGKLCDLDGVKPLILRDCSVAGWLLGNPMCDEFINVPEWSYIVEKANPVNDLCYPGD 110
J57             KTHNGKLCKLNGIPPLELGDCSIAGWLLGNPECDRLLSVPEWSYIMEKENPRDGLCYPGS 109
HK99            TEHNGMLCATSLGHPLILDTCTIEGLVYGNPSCDLLLEGREWSYIVERSSAVNGTCYPGN 112
AI68            SSSTGKICN-NPHRILDGIDCTLIDALLGDPHCDVFQNETWDLFVERSKAFS--NCYPYD 117
SH13            RTNIPRICS-KGKRTVDLGQCGLLGTITGPPQCDQFLEFSADLIIERREGSD--VCYPGK 109
JX13            STGINRLCM-KGRKHKDLGNCHPIGMLIGTPACDLHLTGMWDTLIERENAIA--YCYPGA 108
HB09            SQNLPELCP-SPLRLVDGGQTCDIINGALGSPGCDHLNGAEWDVFIERPNAMD--TCYPFD 113
P10             KEHNGLLCKLKGKAPLDLIDCSLPAWLMGNPKCDELLTASEWAYIKEDPEPENGICFPGD 108
WA79            ITGIDKVCT-KGKKAVDLGSCGILGTIIGPPQCDLHLEFKADLIIERRNSSD--ICYPGR 109
                      :*            *       * * *:          : .        *:*

C09             FIDYEELREQLSSVSSFERFEIFPKTSSWPNHDSNKGVTAACPHAGAKSFYKNLIWLVKK 171
V04             FNDYEELKHLLSRINHFEKIQIIPK-SSWSSHEASLGVSSACPYQGKSSFFRNVVWLIKK 169
J57             FNDYEELKHLLSSVKHFEKVKILPK-DRWTQHTTT-GGSRACAVSGNPSFFRNMVWLTKE 167
HK99            VENLEELRTLFSSASSYQRIQIFPD-------TTWNVTYTGTSRACSGSFYRSMRWLTQK 165
AI68            VPDYASLRSLVASSGTLEFITEGFT---W-TGVTQNGGSNACKRGPGSGFFSRLNWLTKS 173
SH13            FVNEEALRQILRESGGIDKEAMGFT---Y-SGIRTNGATSSCRRS-GSSFYAEMKWLLSN 164
JX13            TVNVEALRQKIMESGGINKISTGFT---YGSSINSAGTTRACMRNGGNSFYAELKWLVSK 165
HB09            VPDYQSLRSILASNGKFEFIAEEFQ---W-TTVKQDGKSGACKRANVNDFFNRLNWLVKS 169
P10             FDSLEDLILLVSNTDHFRKEKIIDM-TRFSDVTTNNVDSACPYDTNGASFYRNLNWV--Q 165
WA79            FTNEEALRQIIRESGGIDKESMGFR---Y-SGIRTDGATSACKRT-VSSFYSEMKWLSSS 164
                  .   *   .                                     .*:  : *:  .

C09             --GNSYPKLSKSYINDKGKEVLVLWGIHHPSTSADQQSIYQNADTYVFVGSSRYSKKFKP 229
V04             --NSTYPTIKRSYNNTNQEDLLVLWGIHHPNDAAEQTKLYQNPTTYISVGTSTLNQRLVP 227
J57             --GSDYPVAKGSYNNTSGEQMLIIWGVHHPIDETEQRTLYQNVGTYVSVGTSTLNKRSTP 225
HK99            --SGFYPVQDAQYTNNRGKSILFVWGIHHPPTYTEQTNLYIRNDTTTSVTTEDLNRTFKP 223
AI68            --GSTYPVLNVTMPNNDNFDKLYIWGIHHPSTNQEQTSLYVQASGRVTVSTRRSQQTIIP 231
SH13            TDNAAFPQMTKSYKNTRKNPALIVWGIHHSGSTAEQTKLYGSGNKLVTVGSSNYQQSFVP 224
JX13            SKGQNFPQTTNTYRNTDTAEHLIMWGIHHPSSTQEKNDLYGTQSLSISVGSSTYRNNFVP 225
HB09            -DGNAYPLQNLTKVNNGDYARLYIWGVHHPSTDTEQTNLYKNNPGGVTVSTKTSQTSVVP 228
P10             --QNKGKQLIFHYQNSENNPLLIIWGVHQTSNAAEQNTYYGSQTGSTTITIGEETNTYPL 223
WA79            MNNQVFPQLNQTYRNTRKEPALIVWGVHHSSSLDEQNKLYGTGNKLITVGSSKYQQSFSP 224
                              *      * :**:*:.    ::   *        :

C09             EIAIRPKVRDQEGRMNYYWTLVEPGDKITFEATGNLVVPRYAFAMERNAG---------S 280
V04             RIATRSKVNGQSGRMEFFWTILKPNDAINFESNGNFIAPEYAYKIVKKGD---------S 278
J57             EIATRPKVNGQGGRMEFSWTLLDMWDTINFESTGNLIAPEYGFKISKRGS---------S 276
```

```
HK99            VIGPRPLVNGLQGRIDYYWSVLKPGQTLRVRSNGNLIAPWYGHVLSGGSH---------G 274
AI68            NIGSRPWVRGLSSRISIYWTIVKPGDVLVINSNGNLIAPRGYFKMRT----------GKS 281
SH13            SPGARTQVNGQSGRIDFHWLMLNPNDTVTFSFNGAFIAPDRASFLR----------GKSM 274
JX13            VVGARPQVNGQSGRIDFHWTLVQPGDNITFSHNGGLIAPSRVSKLI----------GRGL 275
HB09            NIGGRPWVRGQSGRISFYWTIVEPGDLIVFNTIGNLIAPRGHYKLNN---------QKKS 279
P10             VISESSILNGHSDRINYFWGVVNPNQNFSIVSTGNFIWPEYGYFFQKTTN--------IS 275
WA79            SPGARPKVNGQAGRIDFHWMLLDPGDTVTFTFNGAFIAPDRATFLRSNAPSGIEYNGKSL 284
                  .  . :..  .*:.  * ::.  : . .   * :: *     :

C09             GIIISDTPVHDCNTTCQTPKGAINTSLPFQNIHPITIGKCPKYVKSTKLRLATGLRNIPS 340
V04             TIMKSELEYGNCNTKCQTPMGAINSSMPFHNIHPLTIGECPKYVKSNRLVLATGLRNSPQ 338
J57             GIMKTEGTLENCETKCQTPLGAINTTLPFHNVHPLTIGECPKYVKSEKLVLATGLRNVPQ 336
HK99            RILKTDLKSGSCVVQCQTEKGGLNSTLPFHNISKYAFGTCPKYVRVNSLKLAVGLRNVPA 334
AI68            SIMRSDAPIDTCISECITPNGSIPNDKPFQNVNKITYGACPKYVKQNTLKLATGMRNVPE 341
SH13            GIQSGVQVDADCEGDCYYSGGTIISNLPFQNIDSRAVGKCPRYVKQRSLLLATGMKNVPE 334
JX13            GIQSDAPIDNNCESKCFWRGGSINTRLPFQNLSPRTVGQCPKYVNRRSLMLATGMRNVPE 335
HB09            TILNTAIPIGSCVSKCHTDKGSLSTTKPFQNISRIAIGNCPKYVKQGSLKLATGMRNIPE 339
P10             GIIKSSEKISDCDTICQTKIGAINSTLPFQNIHQNAIGDCPKYVKAQELVLATGLRNNPI 335
WA79            GIQSDAQIDESCEGECFYSGGTINSPLPFQNIDSRAVGKCPRYVKQSSLPLALGMKNVPE 344
                 *         *   *    * : .  **:*:   : * **:**.   * ** *::* *

C09             IQ----SRGLFGAIAGFIEGGWTGMVDGWYGYHHQNEQGSGYAADLKSTQNAIDEITNKV 396
V04             RERRRKKRGLFGAIAGFIEGGWQGMVDGWYGYHHSNEQGSGYAADKESTQKAIDGVTNKV 398
J57             IE----SRGLFGAIAGFIEGGWQGMVDGWYGYHHSNDQGSGYAADKESTQKAFDGITNKV 392
HK99            RS----SRGLFGAIAGFIEGGWPGLVAGWYGFQHSNDQGVGMAADRDSTQKAIDKITSKV 390
AI68            K----QTRGLFGAIAGFIENGWEGMIDGWYGFRHQNSEGTGQAADLKSTQAAIDQINGKL 397
SH13            IP---KGRGLFGAIAGFIENGWEGLIDGWYGFRHQNAQGEGTAADYKSTQSAIDQITGKL 391
JX13            LI---QGRGLFGAIAGFLENGWEGMVDGWYGFRHQNAQGTGQAADYKSTQAAIDQITGKL 392
HB09            K----ASRGLFGAIAGFIENGWQGLIDGWYGFRHQNAEGTGTAADLKSTQAAIDQINGKL 395
P10             KE----TRGLFGAIAGFIEGGWQGLIDGWYGYHHQNSEGSGYAADKEATQKAVDAITTKV 391
WA79            KI---RTRGLFGAIAGFIENGWEGLIDGWYGFRHQNAQGQGTAADYKSTQAAIDQITGKL 401
                       **********:*.** *:: ****::*.* :* * *** .:** *.* :. *:

C09             NSVIEKMNTQFTAVGKEFNHLEKRIENLNKKVDDGFLDIWTYNAELLVLLENERTLDYHD 456
V04             NSIIDKMNTQFEAVGREFNNLERRIENLNKKMEDGFLDVWTYNAELLVLMENERTLDFHD 458
J57             NSVIEKMNTQFEAVGKEFGNLERRLENLNKRMEDGFLDVWTYNAELLVLMENERTLDFHD 452
HK99            NNIVDKMNKQYEIIDHEFSEVETRLNMINNKIDDQIQDVWAYNAELLVLLENQKTLDEHD 450
AI68            NRVIEKTNEKFHQIEKEFSEVEGRIQDLEKYVEDTKIDLWSYNAELLVALENQHTIDLTD 457
SH13            NRLIEKTNQQFELIDNEFTEVEKQIGNVINWTRDSITEVWSYNAELLVAMENQHTIDLAD 451
JX13            NRLVEKTNTEFESIESEFSEIEHQIGNVINWTKDSITDIWTYQAELLVAMENQHTIDMAD 452
HB09            NRLIEKTNEKYHQIEKEFEQVEGRIQDLEKYVEDTKIDLWSYNAELLVALENQHTIDVTD 455
P10             NNIIDKMNTQFESTAKEFNKIEMRIKHLSDRVDDGFLDVWSYNAELLVLLENERTLDFHD 451
WA79            NRLIEKTNKQFELIDNEFTEVEQQIGNVINWTRDSLTEIWSYNAELLVAMENQHTIDLAD 461
                * :::* * ::     ** .:* ::  : .   *   ::*:*:***** :**::*:*  *

C09             SNVKNLYEKVRSQLKNNAKEIGNGCFEFYHKCDNTCMESVKNGTYDYPKYSEEAKLNREE 516
V04             SNVKNLYDKVRLQLRDNAKELGNGCFEFYHKCDNECMESVRNGTYDYPQYSEEARLKREE 518
J57             SNVKNLYDKVRMQLRDNVKELGNGCFEFYHKCDDECMNSVKNGTYDYPKYEEESKLNRNE 512
HK99            ANVNNLYNKVKRALGSNAMEDGKGCFELYHKCDDQCMETIRNGTYNRRKYREESRLERQK 510
AI68            SEMNKLFEKTRRQLRENAEEMGNGCFKIYHKCDNACIESIRNGTYDHDVYRDEALNNRFQ 517
SH13            SEMDKLYERVKRQLRENAEEDGTGCFEIFHKCDDDCMASIRNNTYDHSKYREEAMQNRIQ 511
JX13            SEMLNLYERVRKQLRQNAEEDGKGCFEIYHACDDSCMESIRNNTYDHSQYREEALLNRLN 512
HB09            SEMNKLFERVRRQLRENAEDKGNGCFEIFHKCDNNCIESIRNGTYDHDIYRDEAINNRFQ 515
P10             ANVNNLYQKVKVQLKDNAIDMGNGCFKILHKCNNTCMDDIKNGTYNYYEYRKESHLEKQK 511
```

```
WA79            SEMNKLYERVRRQLRENAEEDGTGCFEIFHRCDDQCMESIRNNTYNHTEYRQEALQNRIM 521
                ::: :*:::.:  * .*. : *.***:: * *:: *:  ::*.**:   * .*:  ::

C09             IDGVKLEST 525
V04             ISGVKLESI 527
J57             IKGVKLSSM 521
HK99            IEGVKLESE 519
AI68            IKGVELKSG 526
SH13            IDPVKLSS- 519
JX13            INPVTLSS- 520
HB09            IQGVKLTQG 524
P10             IDGVKLSEN 520
WA79            INPVKLSS- 529
                *. * * .
```

```
Multiple Sequence Alignment of Coronavirus RBDs


SARS-2          VPSGDVVRFPNITNLCPFGEVFNATKFPSVYAWERKKISNCVADYSVLYNST-FFSTFKC  59
RaTG13          SPVTEVVRFPNITNLCPFDKVFNATRFPSVYAWERTKISDCVADYTVFYNST-SFSTFNC  59
SHC014          LPSTEVVRFPNITNFCPFDKVFNATRFPNVYAWQRTKISDCIADYTVLYNST-SFSTFKC  59
Rs4081          SPSTEVIRFPNITNRCPFDRVFNASRFPSVYAWERTKISDCVADYTVLYNST-SFSTFKC  59
Pang17          APSKEVVRFPNITNLCPFGEVFNATTFPSVYAWERKRISNCVADYSVLYNST-SFSTFKC  59
RmYN02          APSKEVVRFPNITNLCPFGEVFNATTFPSVYAWERKRISNCVADYSVLYNST-SFSTFKC  59
Rf1             SPSTEVVRFPNITNLCPFGQVFNASNFPSVYAWERLRISDCVADYAVLYNSSSSFSTFKC  60
WIV1            SPTHEVVRFPNITNRCPFDKVFNASRFPNVYAWERTKISDCVADYTVLYNST-SFSTFKC  59
SARS            QPTESIVRFPNITNLCPFGEVFNATRFASVYAWNRKRISNCVADYSVLYNSA-SFSTFKC  59
Yun11           QPTDSIVRFPNITNLCPFGEVFNATTFASVYAWNRKRISNCVADYSVLYNST-SFSTFKC  59
BM4831          QPTISIVRFPNITNLCPFGEVFNASKFASVYAWNRKRISNCVADYSVLYNST-SFSTFKC  59
BtKY72          TPTTEVVRFPNITQLCPFNEVFNITSFPSVYAWERMRITNCVADYSVLYNSSASFSTFQC  60
                 *  .::******: ***..*** : *..****:* :*::*:***:*:***:  ****:*

SARS-2          YGVSATKLNDLCFSNVYADSFVVKGDDVRQIAPGQTGVIADYNYKLPDDFMGCVLAWNTR 119
RaTG13          YGVSPSKLIDLCFTSVYADTFLIRFSEVRQVAPGQTGVIADYNYKLPDDFTGCVIAWNTA 119
SHC014          YGVSPSKLIDLCFTSVYADTFLIRFSEVRQIAPGETGVIADYNYKLPDDFTGCVLAWNTA 119
Rs4081          YGVSPSKLIDLCFTSVYADTFLIRFSEVRQIAPGETGVIADYNYKLPDEFTGCVIAWNTA 119
Pang17          YGVSATKLNDLCFSNVYADSFVVKGDDVRQIAPGQTGVIADYNYKLPDDFLGCVLAWNTN 119
RmYN02          YGVSATKLNDLCFSNVYADSFVVKGDDVRQIAPGQTGVIADYNYKLPDDFTGCVLAWNTR 119
Rf1             YGVSPTKLNDLCFSSVYADYFVVKGDDVRQIAPAQTGVIADYNYKLPDDFTGCVLAWNTN 120
WIV1            YGVSPSKLIDLCFTSVYADTFLIRSSEVRQVAPGETGVIADYNYKLPDDFTGCVIAWNTA 119
SARS            YGVSPTKLNDLCFTNVYADSFVIRGDEVRQIAPGQTGKIADYNYKLPDDFTGCVIAWNSN 119
Yun11           YGVSPTKLNDLCFTNVYADSFVITGDEVRQIAPGQTGKIADYNYKLPDDFTGCVIAWNSK 119
BM4831          YGVSPTKLNDLCFTNVYADSFVVKGDEVRQIAPGQTGVIADYNYKLPDDFTGCVIAWNSV 119
BtKY72          YGVSPTKLNDLCFSSVYADYFVVKGDDVRQIAPAQTGVIADYNYKLPDDFTGCVIAWNTN 120
                ****.:** ****:.**** *::  .:***:**.:** **********:* ***:***: 

SARS-2          NIDATSTGNYNYKYRYLRHGKLRPFERDISNVPFSPDGKPCTP-PALNCYWPLNDYGFYT 178
RaTG13          KQDVGS-----YFYRSHRSSKLKPFERDLSSE--------------ENGVRTLSTYDFNQ 160
SHC014          QQDIGS-----YFYRSHRAVKLKPFERDLSSD--------------ENGVRTLSTYDFNP 160
Rs4081          NQDRGQ-----YYYRSSRKTKLKPFERDLSSD--------------ENGVRTLSTYDFYP 160
Pang17          SKDSSTSGNYNYLYRWVRRSKLNPYERDLSNDIYSPGGQSCSA-VGPNCYNPLRPYGFFT 178
RmYN02          NIDATQTGNYNYKYRSLRHGKLRPFERDISNVPFSPDGKPCTP-PAFNCYWPLNDYGFYI 178
Rf1             SVDSKSGNN--FYYRLFRHGKIKPYERDISNVLYNSAGGTCSSISQLGCYEPLKSYGFTP 178
WIV1            KQDQGQ-----YYYRSSRKTKLKPFERDLTSD--------------ENGVRTLSTYDFYP 160
SARS            NLDSKVGGNYNYLYRLFRKSNLKPFERDISTEIYQAGSTPCNGVEGFNCYFPLQSYGFQP 179
Yun11           HIDAKEGGNFNYLYRLFRKANLKPFERDISTEIYQAGSKPCNGQTGLNCYYPLYRYGFYP 179
BM4831          KQDALTGGNYGYLYRLFRKSKLKPFERDISTEIYQAGSTPCNGQVGLNCYYPLERYGFHP 179
BtKY72          SLD--SSNE--FFYRRFRHGKIKPYGRDLSNVLFNPSGGTCSA-EGLNCYKPLASYGFTQ 175
                  *        : **  *  ::.*: **::.                .   .*  *.*

SARS-2          TTGIGYQPYRVVVLSFELLNAPATVCGPKLSTDLIKNQCVNFNFNGLTGTGVLTPSSKRF 238
RaTG13          NVPLEYQATRVVVLSFELLNAPATVCGPKLSTSLVKNQCVNFNFNGFKGTGVLTDSSKTF 220
SHC014          NVPLDYQATRVVVLSFELLNAPATVCGPKLSTQLVKNRCVNFNFNGLRGTGVLTDSDKRF 220
Rs4081          SVPLEYQATRVVVLSFELLNAPATVCGPKLSTSLIKNQCVNFNFNGLKGTGVLTDSSKKF 220
Pang17          TAGVGHQPYRVVVLSFELLNAPATVCGPKLSTDLIKNQCVNFNFNGLTGTGVLTPSSKRF 238
RmYN02          TNGIGYQPYRVVVLSFELLNAPATVCGPKLSTDLIKNQCVNFNFNGLTGTGVLTPSSKRF 238
Rf1             TVGVGYQPYRVVVLSFELLNAPATVCGPKKSTELVKNKCVNFNFNGLTGTGVLTSSTKKF 238
WIV1            NVPIEYQATRVVVLSFELLNAPATVCGPKLSTALVKNQCVNFNFNGLKGIGVLTDSSKRF 220
SARS            TNGVGYQPYRVVVLSFELLHAPATVCGPKKSTNLVKNKCVNFNFNGLTGTGVLTESNKKF 239
```

```
Yun11               TDGVGHQPYRVVVLSFELLNAPATVCGPKKSTNLVKNKCVNFNFNGLTGTGVLTESNKKF 239
BM4831              TTGVNYQPFRVVVLSFELLNGPATVCGPKLSTTLVKDKCVNFNFNGLTGTGVLTTSKKQF 239
BtKY72              SSGIGFQPYRVVVLSFELLNAPATVCGPKQSTELVKNKCVNFNFNGLTGTGVLTNSTKKF 235
                    .  : .*. **********:.******** ** *:*::********: * **** * * *

SARS-2              QPFQQFGRDVSDFTDSVRDPKTSEILDISPCSF 271
RaTG13              QSFQQFGRDASDFTDSVRDPQTLRILDISPCSF 253
SHC014              QSFQQFGRDSADFTDSVRDPQTLQILDISPCSF 253
Rs4081              QSFQQFGRDASDFTDSVRDPQTLQILDISPCSF 253
Pang17              QPFQQFGRDVSDFTDSVRDPKTSEILDISPCSF 271
RmYN02              QPFQQFGRDVSDFTDSVRDPKTSEILDISPCSF 271
Rf1                 QPFQQFGRDVSDFTDSVRDPKTFEILDISPCSY 271
WIV1                QSFQQFGRDTSDFTDSVRDPQTLQILDITPCSF 253
SARS                LPFQQFGRDIADTTDAVRDPQTLEILDITPCSF 272
Yun11               LPFQQFGRDIADTTDAVRDPQTLEILDITPCSF 272
BM4831              LPFQQFGRDISDTTDAVRDPQTLEILDITPCSF 272
BtKY72              QPFQQFGRDVSDFTDSVRDPKTLEILDIAPCSY 268
                     .******* :* **:****:* .****:***:
```
